# A review of metal-organic frameworks and polymers in mixed matrix membranes for CO_2_ capture

**DOI:** 10.3762/bjnano.16.14

**Published:** 2025-02-12

**Authors:** Charlotte Skjold Qvist Christensen, Nicholas Hansen, Mahboubeh Motadayen, Nina Lock, Martin Lahn Henriksen, Jonathan Quinson

**Affiliations:** 1 Department of Biological and Chemical Engineering, Aarhus University, Ole Worms Allé 3, 8000 Aarhus C, Denmark; 2 Centre for Water Technology (WATEC), Aarhus University, Ole Worms Allé 3, 8000 Aarhus C, Denmark; 3 Interdisciplinary Nanoscience Center (iNANO), Aarhus University, Gustav Wieds Vej 14, 8000 Aarhus C, Denmark; 4 Department of Electrical and Computer Engineering, Aarhus University, Finlandsgade 22, 8200 Aarhus N, Denmark; 5 Department of Biological and Chemical Engineering, Aarhus University, Aabogade 40, 8200 Aarhus N, Denmark

**Keywords:** CO_2_ capture, gas separation, inorganic filler, metal-organic framework (MOF), mixed matrix membrane (MMM)

## Abstract

Polymeric membranes offer an appealing solution for sustainable CO_2_ capture, with potential for large-scale deployment. However, balancing high permeability and selectivity is an inherent challenge for pristine membranes. To address this challenge, the development of mixed matrix membranes (MMMs) is a promising strategy. MMMs are obtained by carefully integrating porous nano-fillers into polymeric matrices, enabling the simultaneous enhancement of selectivity and permeability. In particular, metal-organic frameworks (MOFs) have gained recognition as MMM fillers for CO_2_ capture. Here, a review of the current state, recent advancements, and challenges in the fabrication and engineering of MMMs with MOFs for selective CO_2_ capture is proposed. Key considerations and promising research directions to fully exploit the gas separation potential of MOF-based MMMs in CO_2_ capture applications are highlighted.

## Review

### 1 Introduction

The continuous rise in global CO_2_ emissions has unfolded an era of unprecedented climate change with profound ecological and environmental consequences [1]. Therefore, the urgency of mitigating the environmental impact of elevated CO_2_ levels raises a strong motivation to achieve large-scale reduction of CO_2_ emissions [2]. In this context, CO_2_ capture processes have received much attention [3–4].

Considerable research has been dedicated to enhancing the efficiency of CO_2_ capture technologies for large-scale applications, particularly in natural gas purification and post-combustion processes [5]. Various technologies are currently under investigation for the capture of CO_2_, including regenerative solvent-based absorption [2,6], fixed-bed adsorption [7], cryogenic separation techniques [8], and membrane separation methods [9–12]. Of these, membrane technology offers advantages such as exceptional stability, high efficiency, low energy consumption, and ease of operation [5]. However, a significant drawback of membrane separation is the inherent trade-off between permeability (pressure-normalized flux) and selectivity (α_A/B_) for gases A and B, as described by the relationship in Equation 1 [5,12–14].


[1] $$P_{\text{A}}=k\cdot \alpha_{\text{A}/\text{B}}^{n},$$

where *P*_A_ is the steady-state permeability of the more permeable gas A [Barrer], α is the selectivity for gas A over gas B (*P*_A_/*P*_B_), and *k* and *n* are gas pair-specific constants, that is, *k* is the pre-factor [Barrer], and *n* is the slope of the trade-off relationship, which is typically negative. Extensive research efforts in the membrane separation field aim to improve CO_2_ permeability and selectivity to enhance the efficiency of CO_2_ capture. A promising approach in this field involves hybrid polymer composite membranes known as mixed matrix membranes (MMMs) [15–22]. Among the diverse range of inorganic fillers integrated into MMMs, metal-organic frameworks (MOFs) have received a growing focus in the past decade [5,12,23–25]. The MOFs play the role of versatile and porous dispersive fillers, providing a multitude of opportunities to fabricate highly efficient MOF-derived MMMs tailored for CO_2_ separation.

**Figure 1:**
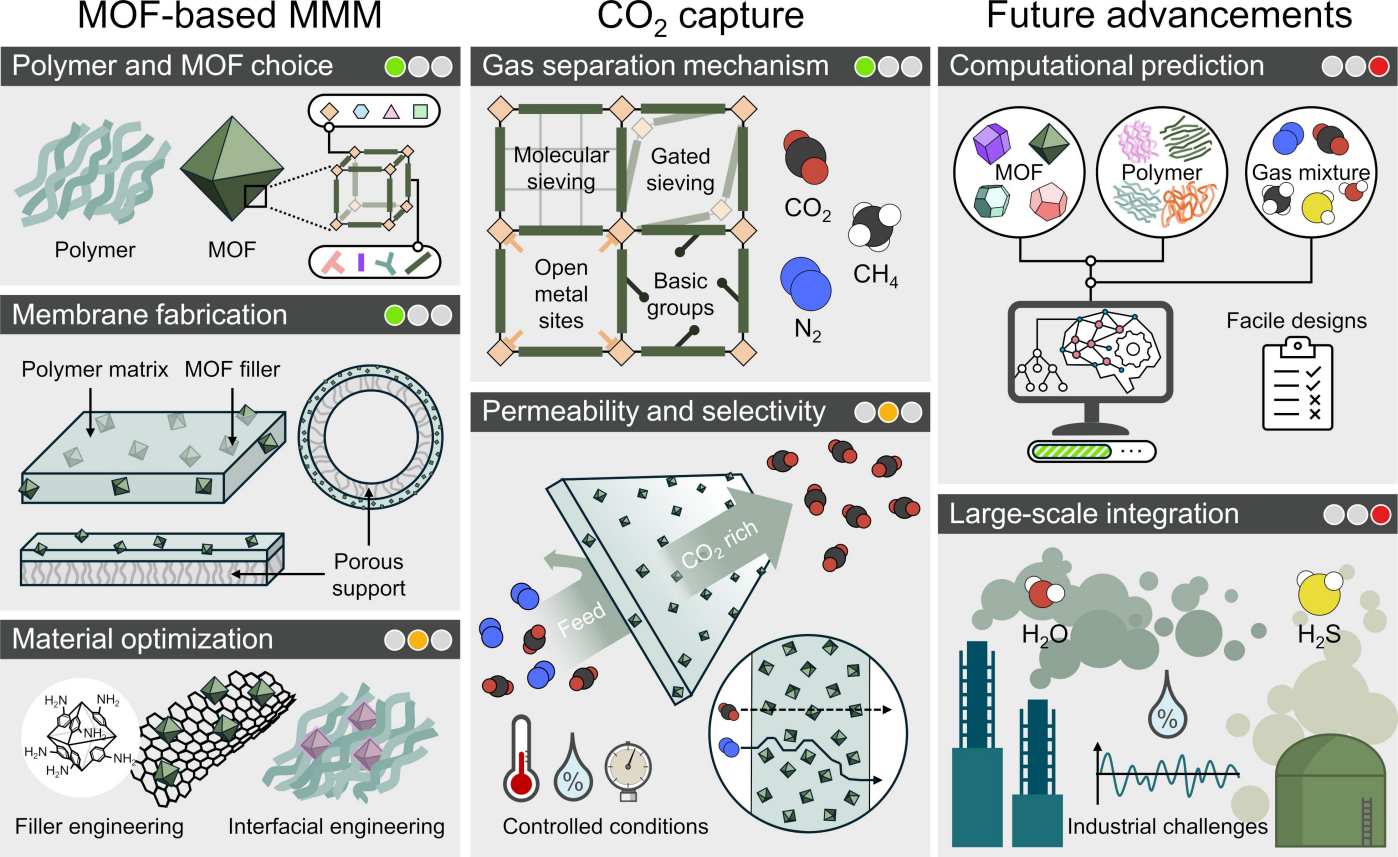
Overview of subtopics covered in this review for the key research areas related to MOF-based MMMs for CO_2_ capture. Colored circles indicate the maturity levels from established (green) to developing (yellow) and emerging (red) research areas. “Polymer and MOF choice” is discussed in sections 3.2 and 3.4 through 4.1, “Membrane fabrication” in section 3.3, “Material optimization” in sections 3.4 through 4.2, “Gas separation mechanism” in section 2, “Permeability and selectivity” in sections 2, 4.2, and 6.2, “Computational prediction” in section 5, and “Large-scale integration” in section 6.

The present review aims to provide an introductory overview of relevant aspects in the field of CO_2_ capture by MOF-based MMMs and is articulated around the subtopics illustrated in Figure 1. First, a broad introduction to this area of research is provided. Following, the focus is shifted to specific challenges and opportunities encountered in the development and fabrication of MOF-based MMMs for CO_2_ capture. An account of current trends in the field is given, while gaps and further areas of investigation are identified and highlighted. Specifically, the review intends to convey a broad yet comprehensive understanding of (1) mechanisms of CO_2_ sorption in MOFs, (2) considerations related to the integration of MOFs in MMMs, (3) CO_2_ capture performance of MOF-based MMMs, (4) advancements in MOF-based MMM materials design through machine learning, and (5) considerations for the implementation of MOF-based MMMs in large-scale CO_2_ capture applications.

The contents of this review are at the junction of different research areas for which interested readers can refer to dedicated existing literature. For example, Sumida et al. [26] provide a comprehensive review of CO_2_ capture using MOFs, while details on the fabrication of MOF-based MMMs in a general context have been published by Lin et al. [27] and Kitao and coworkers [28]. Focusing on gas separation in general, Daglar et al. [29] have summarized computational approaches to simulate gas transport in thin-film MOF membranes and MOF-based MMMs. With an emphasis on CO_2_ capture specifically, Demir et al. [23] have outlined past and recent advances in MOF-based MMMs for CO_2_ separation with a focus on the influence of the filler, and Rangaraj et al. [5] have comprehensively reviewed the CO_2_ capture performance of various MOF-based MMMs and hybrid technologies reported in the literature.

### 2 MOF-based CO_2_ capture strategies

MOFs are a highly porous subset of crystalline solids [30], first reported by Kinoshita et al. in 1959 [31]. The structural architecture of MOFs consists of inorganic ion nodes coordinated by organic linkers, illustrated in Figure 1. The linkers are rigid and contain at least two functional groups, which allow for their polymerization with the metal ions to form stable and well-defined 3D structures. A plethora of different linkers (e.g., 2,5-dioxido-1,4-benzenedicarboxylic acid (H_2_DOBDC), 1,3,5-benzenetricarboxylic acid (H_3_BTC), and 1,4-benzenedicarboxylic acid (H_2_BDC)) can be combined with various metal ions (e.g., Mg^2+^, Cu^2+^, Zn^2+^, Fe^3+^, and Zr^4+^) to obtain MOFs with diverse structures (e.g., Mg_2_(DOBDC), CuBTC, and Zr_6_O_4_(OH)_4_(BDC)_6_). Moreover, utilizing linkers pre-functionalized with substituents (e.g., –Br, –NH_2_, –SO_3_H) enables straightforward modification of the synthesized MOFs and allows for the synthesis of multifunctional frameworks [32–35]. Alternatively, MOFs can undergo post-synthetic modification to achieve similar functionalization without the risk of degrading functionalized linkers during high-temperature and high-pressure MOF synthesis [32]. The resulting constructions have pore sizes ranging from microporous (<2 nm) to mesoporous (2–50 nm) [30,36]. This simple strategy has led to substantial exploration of various MOFs with different structures and properties for various applications in heterogeneous catalysis, biomedicine, sensing, and gas separation and storage [36–39].

In parallel, the last decade has witnessed a substantial increase in research studies across various fields aimed at mitigating CO_2_ emissions [40–44]. Notably, MOFs have been targeted for CO_2_ capture and separation applications because of their outstanding gas adsorption capabilities [30,45]. The topic of MOF-based gas separation and CO_2_ capture has been extensively addressed in comprehensive reviews [26,46–49]. A succinct account of MOF development for CO_2_ capture is proposed in the next paragraphs to better understand their promising roles as fillers in MMMs.

**Figure 2:**
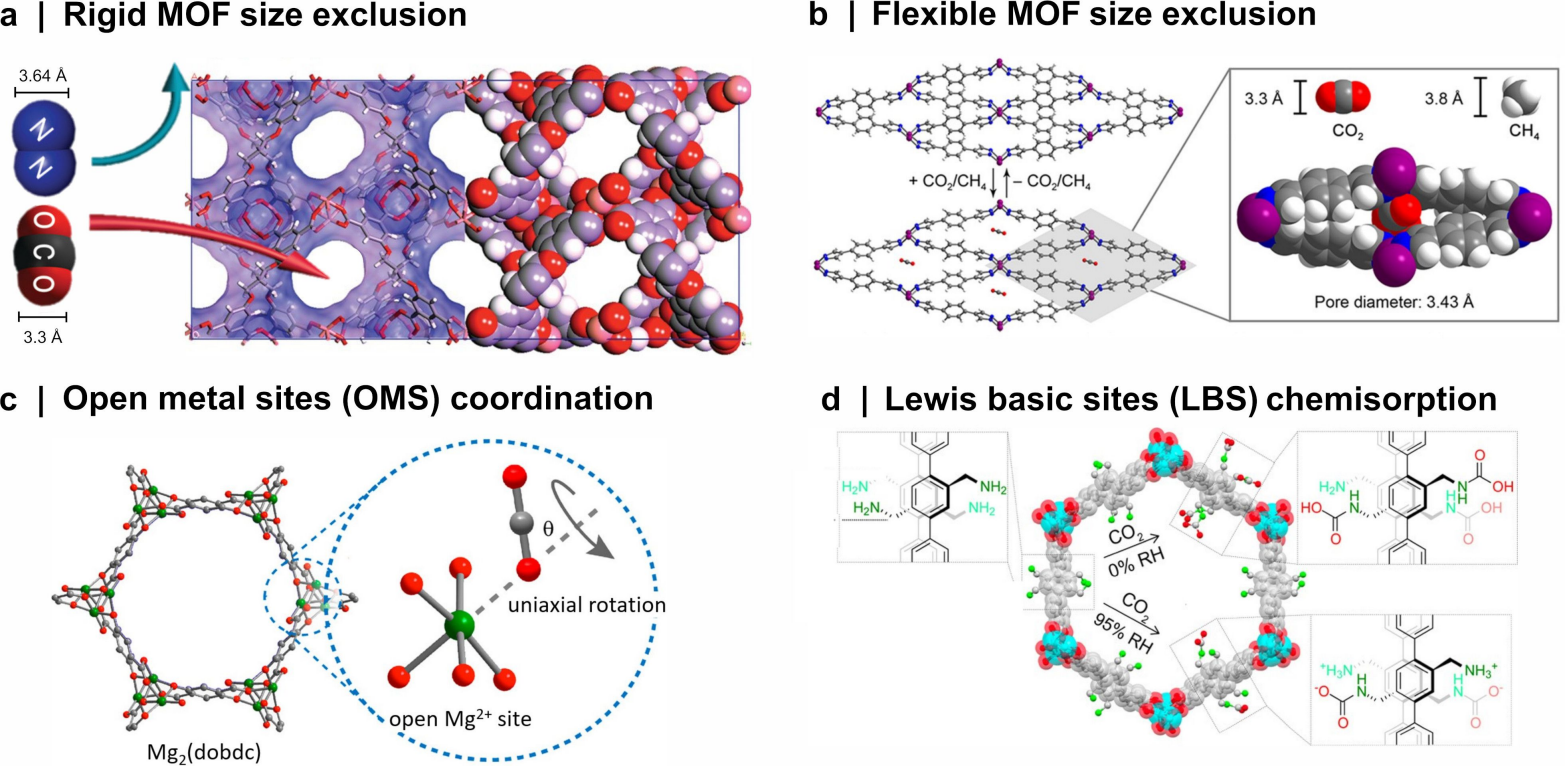
(a) CO_2_ uptake by size exclusion in rigid PCN-29. Adapted with permission from [50], Copyright 2011 American Chemical Society. This content is not subject to CC BY 4.0. (b) CO_2_ uptake by flexible Co(BDP). Adapted with permission from [51], Copyright 2018 American Chemical Society. This content is not subject to CC BY 4.0. (c) CO_2_ coordination to Mg^2+^ in Mg-MOF-74. Adapted with permission from [52], Copyright 2012 American Chemical Society. This content is not subject to CC BY 4.0. (d) CO_2_ chemisorption to Lewis basic sites of IRMOF-74-III-(CH_2_NH_2_)_2_, with possible pore environments before and after exposure to CO_2_ under 95% relative humidity and dry conditions. Adapted with permission from [53], Copyright 2017 American Chemical Society. This content is not subject to CC BY 4.0.

The versatility of MOFs enables the synthesis of CO_2_-selective MOFs with high CO_2_ uptake capacities [23]. By choosing or engineering appropriate metal nodes and organic linkers, the physical and chemical properties of the framework can be tuned to favorably interact with CO_2_, achieving excellent CO_2_ adsorption capacity and preferential CO_2_ adsorption over other gas molecules such as N_2_ or CH_4_. Figure 2 depicts examples of the separation strategies commonly employed in MOF research.

The first approach to promote CO_2_ selectivity involves molecular sieving [54]. This strategy capitalizes on the combination of high surface areas and the tunable nature of pore openings and channels of MOFs, achieving selective CO_2_ adsorption by size exclusion [55], illustrated in Figure 2a. The kinetic diameter of competing adsorbates is the most commonly quoted metric for size comparison, that is, the size of the molecules based on the possibility of collision with other molecules [56]. Consequently, MOFs that exhibit pore sizes around the kinetic diameter of CO_2_ of 3.3 Å effectively allow for CO_2_ separation from gaseous mixtures containing larger molecules such as CH_4_ and N_2_ with kinetic diameters of 3.8 and 3.6 Å, respectively [57]. Recently, Berdichevsky et al. [58] synthesized Zn_3_(NH_2_BDC)_3_(DABCO), a MOF with ultramicropores (3.47 Å pore aperture, 4.90 Å pore width), which was found to be capable of sieving CO_2_ from N_2_ and CH_4_ systems with a very large CO_2_ selectivity. Simulations revealed a CO_2_/N_2_ selectivity of 4800 on a post-combustion flue gas (15/85 mixture of CO_2_/N_2_) and a CO_2_/CH_4_ selectivity of 5 × 10^28^ on a 50/50 CO_2_/CH_4_ mixture, placing this MOF among the theoretically best CO_2_-selective molecular sieves reported to date [58].

Certain MOFs exhibit structural flexibility, which may undergo dynamic changes in response to external stimuli, such as pressure and temperature. This characteristic allows for gated CO_2_ adsorption under specific conditions [44], which presents itself as a unique strategy to selectively control CO_2_ separation in gas mixtures. For instance, Taylor et al. [51] conducted experiments using Co(BDP), a MOF that undergoes structural changes when exposed to both pure CO_2_ at 3.6 bar and a 50/50 mixture of CO_2_/CH_4_ at 7.2 bar. Under these conditions, the one-dimensional channels of the framework expanded to create an aperture ideally suited to adsorb CO_2_ and exclude CH_4_, as depicted in Figure 2b. Above 15 bar pressure, Co(BDP) undergoes expansion to phases with larger pores capable of admitting CH_4_ molecules [59]. However, favorable enthalpic CO_2_ uptake was found to drive the continued exclusion of CH_4_ [51]. In adsorption experiments with 50/50 mixtures of CO_2_/CH_4_ conducted at 7 to 25 bar, it was observed that the amount of CO_2_ adsorbed closely matched the CO_2_ single component isotherm. In contrast, the amount of CH_4_ adsorbed was negligible or close to zero in all cases, indicating near-perfect selectivity for CO_2_. The near-perfect selectivity did not persist when tested in a CO_2_/CH_4_ mixture with a 6/94 molar ratio (α_CO2/CH4_ = 61 ± 4), emphasizing the importance and the challenges related to developing MOF structures for CO_2_ capture [51].

While some MOFs do feature pore apertures within the narrow size range allowing for size exclusion (3.3–3.6 Å), the majority of MOFs exhibiting high adsorption capacities for CO_2_ have pore openings that largely surpass the dimensions of the CO_2_ molecule [26]. Consequently, most current MOF-related studies rely on the separation of the molecules based on the adsorptive interactions between the MOF framework and the CO_2_ adsorbate. A third strategy for obtaining high CO_2_ adsorption selectivity is to prepare MOFs from metal ion nodes that are uncoordinated, thus having accessible coordination vacancies known as open metal sites (OMSs). This strategy, illustrated in Figure 2c, is a prominent method for enhancing CO_2_ capture in MOFs [55]. The formation of OMSs involves solvent removal upon activation, which establishes uncoordinated metal sites capable of adsorbing guest molecules reversibly [60]. A wide range of OMS-bearing MOFs, including NU-1000, MIL-101, and the M-MOF-74 isoreticular series (M = Mg, Ni, Co, and Zn), have been employed as effective CO_2_ adsorbents [44]. The M-MOF-74 series, featuring one-dimensional hexagonal channels with OMSs at the secondary building units (SBUs), has become one of the most extensively studied sets of MOFs for CO_2_ capture. These MOFs have one of the highest densities of OMSs on their channel pore surfaces [61], with a volumetric density of accessible metal sites of 7.5–7.7 mmol·cm^−3^ [62]. The CO_2_ binding strength and selectivity are influenced by the nature of the metal center, with Mg^2+^ ions identified as the preferential adsorption site according to density functional theory (DFT) calculations and supported by neutron powder diffraction experiments and infrared spectroscopy [52,63–65]. It is agreed in literature that CO_2_ molecules bind to Mg^2+^ sites with end-on coordination, forming an angular Mg^2+^·OCO complex, with the rotation angle θ depending on the degree of CO_2_ loading [52,64]. The intramolecular angle of CO_2_ is a matter of debate due to conflicting findings. Neutron diffraction profiles unexpectedly seem to predict large apparent O–C–O bond bending, that is, bond angles of 160–167° [63,66], while theoretical calculations based on DFT only indicate minimal deviations from linear geometry, that is, bond angles of 174–180°. There is a consensus that the interaction of CO_2_ with Mg^2+^ is strongly electrostatic and physisorptive [52,65], with bond strengths in a range that facilitates both efficient CO_2_ capture and effective Mg-MOF-74 regeneration [63].

In addition to targeting adsorption based on the OMSs, organic linkers can also contribute to augmenting the adsorption properties of MOFs. The introduction of Lewis basic sites (LBSs) by ligand modification is one effective approach [44]. This fourth strategy is often implemented by synthesizing amine-functionalized MOFs. The uncoordinated and electron-rich nitrogen atoms in amines are ‘CO_2_-philic’ [33,35] and provide active adsorption sites for CO_2_ through Lewis acid–base interaction between CO_2_ and amines [67]. As illustrated in Figure 2d, adsorptive selectivity can also result from chemical interactions between CO_2_ and LBSs, offering higher selectivity than purely physisorption-related mechanisms [26]. For example, alkylamine-functionalized IRMOF-74-III compounds have been demonstrated to be efficient CO_2_ adsorbents [53,68]. In dry conditions, the alkylamines in IRMOF-74-III-(CH_2_NH_2_)_2_ chemically bind CO_2_ to form new, covalent carbamic acid (RNHCOOH) species at an adsorption capacity of 1.2 mmol CO_2_·g^−1^ [53]. Since the adsorption takes place at the LBSs rather than the OMSs, the influence of humidity was negligible to the adsorption performance. In wet conditions at 95% relative humidity (RH), chemisorption of CO_2_ resulted in ammonium carbamate (RNHCOO^−+^H_3_NR) species formation, and the adsorption capacity remained at 1.2 mmol CO_2_·g^−1^ [53].

### 3 Integration of MOFs in MMMs

#### 3.1 General consideration for MMMs

The concept of MMMs dates back to the 1860s [16,69] and the need to understand the permeability and selectivity properties of membranes. Conventionally, membranes are classified into two categories, namely, organic and inorganic. Organic polymer membranes possess open and flexible structures that facilitates rapid gas diffusion, resulting in excellent permeability but low selectivity. In contrast, inorganic membranes are rigid with small, uniform pores, offering high selectivity but only modest permeability [70]. For both membrane types, increased uniformity in pore size distribution and greater pore rigidity generally enhance selectivity [70–72].

Typically, inorganic membranes exhibit superior thermal, chemical, and mechanical stability compared to organic membranes [72–74]. However, inorganic membranes tend to be brittle, which can lead to crack formation in the finalized membrane, thereby reducing their selective properties. While organic membranes typically possess greater mechanical strength, they are more prone to deformation under mechanical stress [75–76]. Membranes used for gas separation must have sufficient mechanical strength to resist plastic deformation under high gas pressures [77]. Although inorganic membranes appear more advantageous for gas separation, they are typically expensive and challenging to produce on a large scale, whereas organic membranes are cost-effective, simple to manufacture, and reliable for large-scale production [70,72–73].

Consequently, it is desirable to combine the properties of organic and inorganic membranes to balance both membrane permeability and selectivity while achieving better overall stability. This is achieved through the addition of inorganic fillers into organic membrane matrices leading to MMM systems.

Nevertheless, incorporating inorganic additives into organic polymer membranes is complex. Different types of inorganic fillers exhibit unique gas separation properties and interact distinctively with the organic polymer, which itself has distinct gas separation properties [17,75–76,78–79]. Ideally, a high filler loading is desired to push MMM performance boundaries. However, higher filler loadings also increase the likelihood of MOF aggregation (detailed in section 3.5), which may strongly impair the mixed gas separation performance [80]. As a result, achieving the optimal MMM composition is imperative to attain a synergetic effect, leading to the desired case of a design allowing for both excellent separation properties and robust MMM stability.

One of the first MMMs developed with inorganic fillers in the context of the separation of gaseous compounds dates back to 1912 by Steinitzer [81]. Although not denoted as a MMM at the time, the material prepared by Steinitzer is a rubber comprising high amounts of minerals, which in modern terms would be classified as a MMM. The earliest instances of mixed membranes integrating inorganic fillers for gas separation, documented as MMMs, were reported in the late 1980s by Kulprathipanja and coworkers [82]. They developed a silicalite–cellulose acetate MMM with a selectivity of α_CO2/H2_ = 5.15 ± 2.20 on a 50/50 mixture of CO_2_ and H_2_ at 3.45 bar. In comparison, the pristine cellulose acetate membrane exhibited only a selectivity of α_CO2/H2_ = 0.77 ± 0.06, indicating that the incorporation of silicalite in the membrane matrix reversed the selectivity from H_2_ to CO_2_ [82–83]. A few years later in 1992, MMMs were reported in a patent granted to UOP LLC (now Honeywell UOP) [83]. The inventors, Kulprathipanja and coworkers, described the incorporation of inorganic fillers during the fabrication of polymeric membranes with specific permeability. By incorporating fillers with permeability constants similar to that of the polymer membrane and possessing certain adsorbing characteristics towards the target gas, they achieved MMMs with enhanced selectivity and permeability for gas separation.

#### 3.2 MOFs as fillers for MMMs for CO_2_ capture

**Figure 3:**
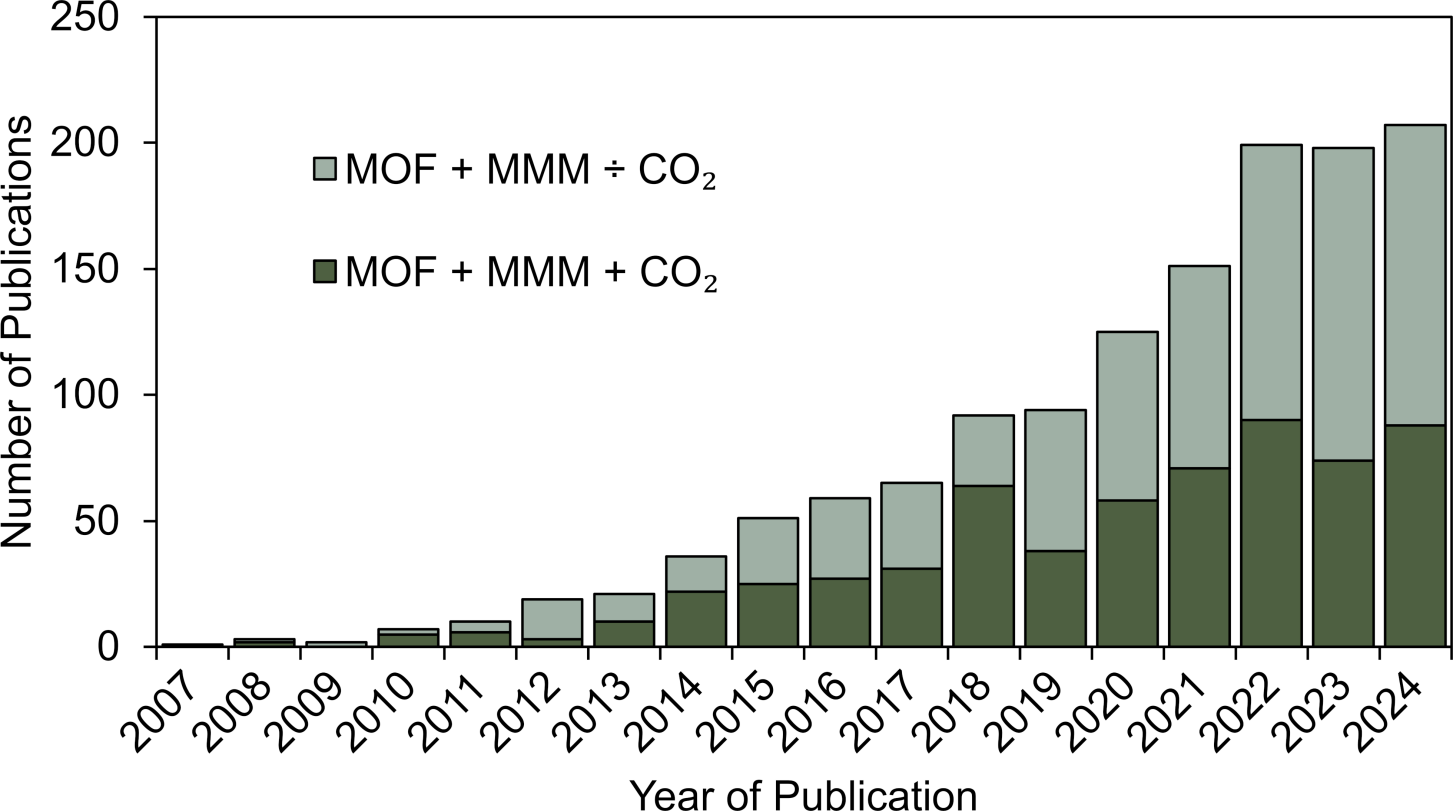
The number of articles related to MOF-derived MMMs in the scientific literature published each year from 2007 to 2023. The bars represent the number of articles returned using the keywords ‘MOF’ or ‘metal organic framework’ and ‘MMM’ or ‘mixed matrix membrane’ in the article title, abstract, or keywords. Dark green bars indicate the number of articles that also contain ‘CO_2_’ in the article title, abstract, or keywords. The Scopus database and search engine were used. Data last updated on October 13, 2024.

Fundamentally, any inorganic additive can be integrated into an organic polymer membrane simply by mixing the inorganic particles with a membrane solution of the specific polymer. This composite solution is then cast to obtain the membrane. However, the general limitation of most inorganic filler-based MMMs is the compatibility between the inorganic filler and the organic membrane. Typically, the organic polymer membrane exhibits low affinity towards the inorganic filler. Accordingly, this severely limits the potential range of the different workable composites [84]. Therefore, large efforts have been made to identify new additive materials more compatible with organic polymer membranes. In this search, MOFs were shown to be promising candidates. The first report on MOF-integrated MMMs was by Yehia et al. in 2004 [85]. In this pioneering work, the authors incorporated the MOF copper(II) biphenyl dicarboxylate-triethylenediamine into poly(3-acetoxyethylthiophene) to enhance the selectivity towards CH_4_. This initial study was followed by an increasing interest in using MOFs as fillers for MMMs. This is reflected by the drastic increase in yearly publications related to MOF-integrated MMMs throughout the past two decades, reported in Figure 3. Many of these studies focus on CO_2_ capture. The key benefit of using MOFs in MMMs rather than simple inorganic particles is their unusually high surface areas with adjustable pore sizes and unique functionalization opportunities [86]. As described in section 2, this allows for exceptional gas adsorption capabilities. Furthermore, MOFs are inherently more compatible with the organic polymer matrix than regular inorganic fillers, as they have organic linkers as a fundamental structural component. Specifically, utilizing a linker with similar chemistry to the membrane polymer and its functional groups can drastically enhance MOF-membrane polymer compatibility [24]. Last, the rich MOF chemistry and tunability pave the way to advanced MMMs with specialized functionality for CO_2_ separation.

For MOF-integrated MMM fabrication, the MOF–polymer matrix interface is crucial to its CO_2_ separation performance (as detailed further in section 3.5). Although the organic linkers within the MOF typically offer support to the membrane polymer matrix, the interface between the MOF and the matrix may still be suboptimal. In this case, the CO_2_ separation performance of the finalized MOF-based MMM may be considerably impaired compared to that of the pristine MOF [86]. The major issue arising from such incompatibility is the formation of void defects within the MMM due to insufficient adhesion between the MOF interface and the polymer matrix. Such voids act as non-specific permeation sites [80]. Consequently, the interfacial compatibility between the MOF and the MMM must be accounted for and optimized when designing and synthesizing the membranes.

#### 3.3 Flat sheet and hollow fiber configurations for MOF-based MMMs

**Figure 4:**
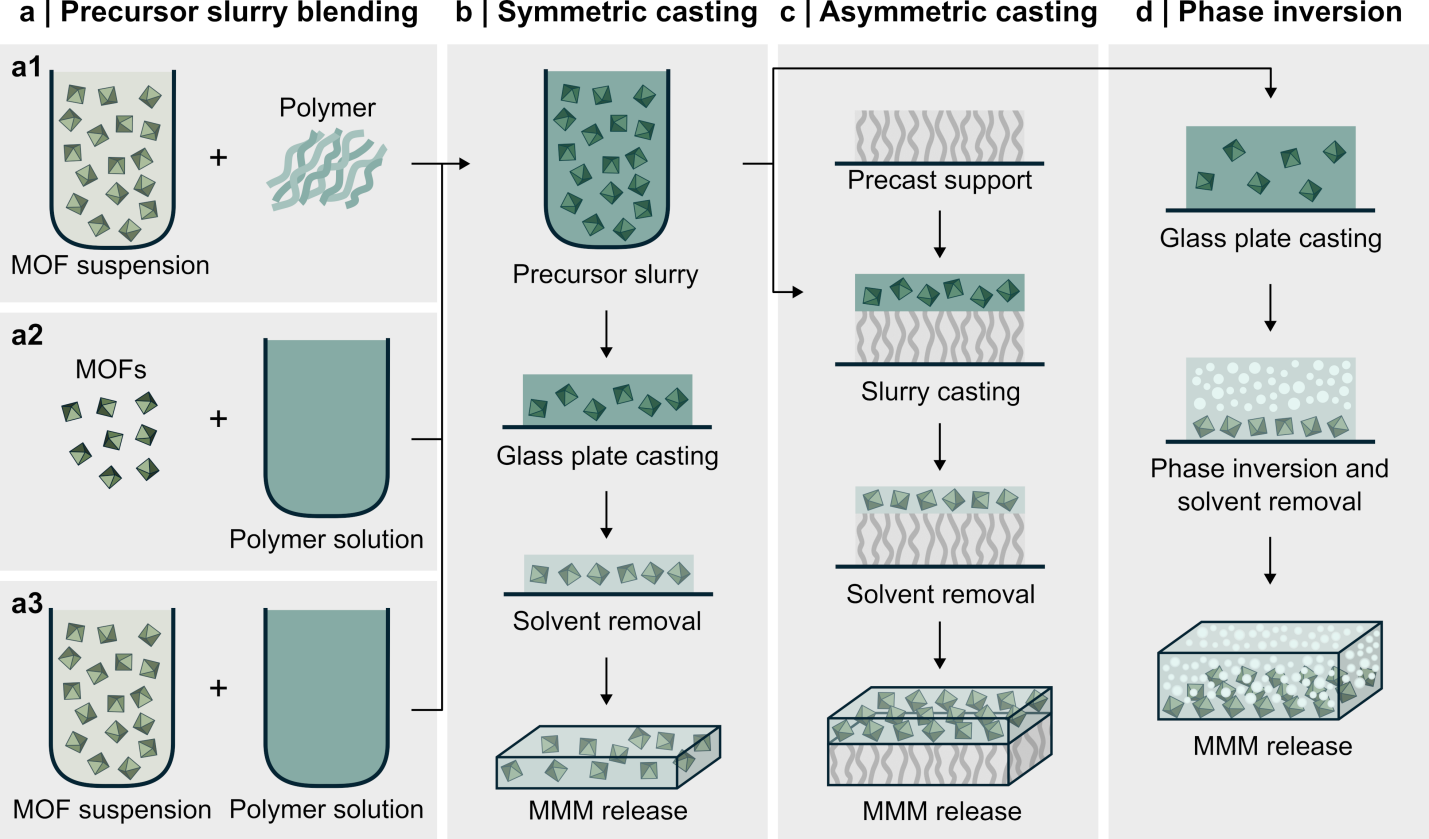
Illustration of different flat sheet MOF-based MMM preparation methods. (a) Different ways of preparing the MOF-based MMM precursor slurry. (b) Solvent casting process for symmetric MOF-based MMMs. (c) Solvent casting process of asymmetric MOF-based MMMs. (d) Phase inversion method for creating asymmetric MOF-based MMMs. Specifically, phase inversion is induced by a conditional change such as temperature.

MOF-based MMMs are fabricated in several configurations, and the most common ones are illustrated in Figure 1. Flat sheet membranes, composed of simple homogeneous or heterogeneous porous sheets, are widely used because of their straightforward design and ease of fabrication [80,87], allowing for compound separation through plain membrane diffusion [88]. Figure 4 illustrates the process of preparing flat sheet MOF-based MMMs through various techniques. Conventionally, flat-sheet MOF-based MMMs are prepared by casting a precursor slurry of the membrane polymer with well-dispersed MOF particles onto a glass plate or support substrate [87,89]. As seen in Figure 4a, the precursor mixture can be prepared by (1) adding the organic polymer to a suspension containing the MOF filler, (2) adding the MOF particles to a solution of the organic polymer, or (3) preparing a polymer solution and a MOF suspension independently and subsequently blending the two [80]. For the third method, priming the MOF suspension with a small amount of polymer before blending with the polymer solution can help reduce MOF–polymer interface defects [80,90–93]. Thorough mixing of the precursor slurry is critical to ensure a homogenous final membrane with MOFs evenly dispersed throughout the polymer matrix. For more detailed information on general MMM fabrication, the reader is referred to the extensive review by Aroon and coworkers [80].

Once the precursor slurry has been prepared, it can be cast into a solid membrane. Flat sheet MMMs can be prepared as symmetric, Figure 4b, or asymmetric membranes, Figure 4c,d. In symmetric membranes, the MMM spans the entire sheet, which imposes mechanical instability for sheets thinner than 50 µm and limits gas flux through the membrane [87]. Asymmetric membranes feature a thin MMM layer supported by a porous polymer layer that provides mechanical stability and high gas flux better suited for industrial applications [80,87]. Both membrane types are typically prepared through simple solution casting. For symmetric membranes, the precursor slurry is cast in a thick layer on a rigid flat substrate, such as a glass or Teflon^®^ petri dish. For asymmetric membranes, the slurry is cast in a thin layer atop a polymer support. Solvent removal following casting yields the finalized MOF-based MMM. This casting method is simple and cost-effective but is primarily relevant in small-scale applications. Asymmetric membranes can alternatively be prepared via phase inversion, where the precursor slurry undergoes phase separation under altered solvent, composition, or temperature conditions to form a porous support phase and a dense MMM phase [80,87,92,94]. Finally, fabrication of thin selective MMM layers on top of porous substrates can be accomplished via spin coating [95].

Defects and non-uniform MOF dispersion formed during casting may strongly impact the functional performance of the MOF-based MMM. Consequently, meticulous preparation of the precursor slurry and precise MMM casting is critical to avoid such issues. Furthermore, the solvents used for the polymer slurry must be sufficiently compatible with the MOF and the membrane polymer employed to achieve optimal MMM products [94].

The solvent dependency in MMM casting has been extensively studied and is well illustrated through the work of Kulak and coworkers [96]. The authors assessed the solvent effect on the final MOF-integrated MMM. Specifically, they used oxolane, dichloromethane, chloroform, and methyl ethyl ketone (MEK) to prepare their precursor slurry. The MMMs were synthesized with 6FDD co-polyimide and MOF-808 at 0, 10, and 30 wt % loadings. Generally, the choice of solvent affects parameters such as viscosity, MOF dispersion, and MOF stabilization in the suspension. The authors found that dichloromethane and chloroform resulted in homogenous MOF dispersion, whereas MEK gave poor MOF dispersion, and oxolane gave intermediate results. Across all MOF loadings tested, they observed that MMMs produced in MEK displayed severe curling and exhibited poor MOF distributions, attributed to the prolonged evaporation times required, as well as the low viscosity and density of this solvent. In contrast, the MMMs produced in oxolane, dichloromethane, and chloroform led to generally well-formed MMMs with reasonable mechanical stability, MOF distribution, and gas separation performance. Another important result from this study is that the solvent effects on MMM formation depend on MOF loading. This was illustrated well through the MOF-based MMMs in dichloromethane, where 30 wt % MOF formed a brittle MMM, whereas the MMM was well-formed with 10 wt % MOF in the same solvent. Moreover, they found that the characteristics of the MMMs formed in the different solvents were reflected in the CO_2_/CH_4_ gas separation performance. With a MOF loading of 10 wt %, all MMMs showed increased permeability compared to the pristine membrane. However, for the oxolane and MEK MMMs, this came at the expense of decreased selectivity due to defects. The dichloromethane and chloroform MMMs showed little to no such detrimental effects [96].

**Figure 5:**
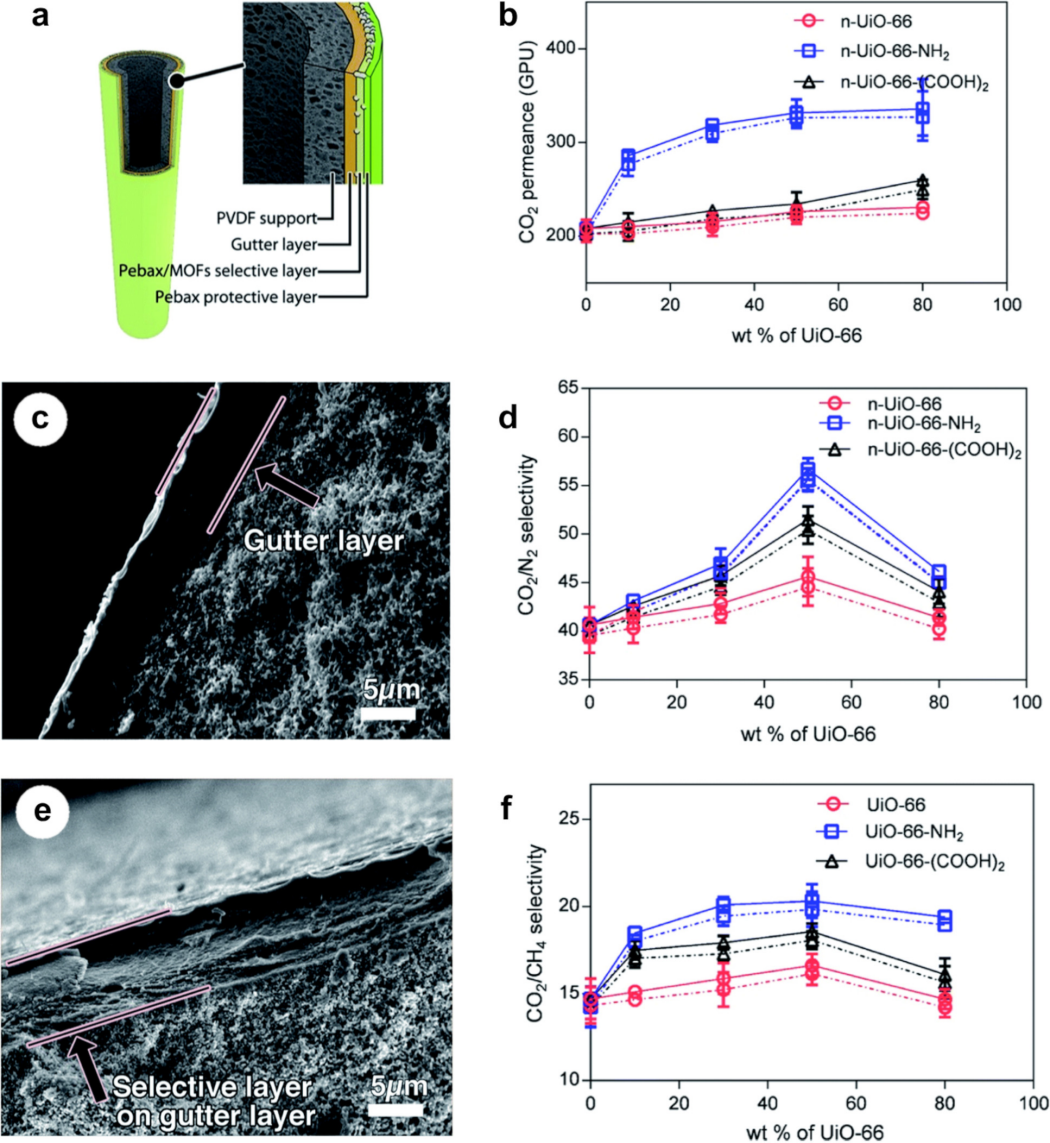
(a) Schematic diagram of a composite hollow fiber mixed matrix membrane. (c, e) Cross-sectional scanning electron microscopy images of composite membranes with (c) PTMSP and (e) PTMSP and pure Pebax^®^ coating layers. (b, d, f) Gas separation performance of UiO-66/Pebax^®^ 1657-based composite membranes at different particle loadings, showing both pure gas (solid lines) and mixed gas (dashed lines) data: (b) CO_2_ permeance, (d) CO_2_/N_2_ gas selectivity, and (f) CO_2_/CH_4_ gas selectivity [98]. Used with permission of The Royal Society of Chemistry, from [98] (“Surface functionalized UiO-66/Pebax-based ultrathin composite hollow fiber gas separation membranes” by P. D. Sutrisna et al., J. Mater. Chem. A, vol. 6, issue 3, © 2018); permission conveyed through Copyright Clearance Center, Inc. This content is not subject to CC BY 4.0.

In the past decade, the fabrication of asymmetric MOF-based MMMs has gained more interest in the scientific CO_2_ capture community as the field has matured and the demand for scalable solutions has increased [97–102]. Asymmetric MOF-based MMMs can reach a MMM layer thickness below 1 µm due to a thick non-selective structural support layer [24], see Figure 4c. Hollow fiber MMMs (HFMMMs) are special types of asymmetric membranes that have become attractive because of their intrinsic high surface-area-to-volume ratio, making them particularly well suited for industrial gas separation [87,103]. HFMMMs are cylindrical or capillary-shaped membranes with internal and exterior diameters smaller than 0.25 and 1.00 mm, respectively [93], see Figure 5 or Figure 1 for structural schematics. These membranes can withstand operating pressures up to 70 bar [24]. HFMMMs are typically prepared through a spinning process followed by a post-treatment solvent exchange [79,97], in which two or more layers are formed, decreasing the mass transfer resistance and increasing the gas permeation flux. Sutrisna et al. [98] fabricated a novel HFMMM consisting of an inner polyvinylidenfluorid (PVDF) porous support dip-coated with a highly permeable poly(1-trimethylsilyl-1-propyne) (PTMSP) gutter layer, a Pebax^®^ 1657-based selective layer containing UiO-66, and a top pure Pebax^®^ 1657 protective layer, illustrated in Figure 5. The thickness of the Pebax^®^ layers reached 1.0–1.5 μm, and the tensile strength of the MOF-loaded membrane was comparable to that of the composite membrane with a pure Pebax^®^ layer. High loadings of up to 50 wt % UiO-66 and its functionalized derivatives (UiO-66-NH_2_ and UiO-66-(COOH)_2_) were achieved without MOF agglomeration in the thin, selective layers. When successfully prepared, these novel HFMMMs can express improved separation efficiency, increased mechanical strength, and improved resistance to temperature and chemical factors over conventional HFMMMs [60,99]. Compared with traditional flat sheet MMMs, the significant decrease in the thickness of the dense, selective layer inherently decreases mass transfer resistance and enhances gas permeation flux [80,87], rendering the HFMMM configuration a promising candidate to be upscaled for CO_2_ capture purposes.

#### 3.4 Considerations for MOF–polymer pairs

In recent years, many CO_2_-selective MOFs have emerged and been combined with numerous polymer matrixes to form CO_2_-selective MMMs. However, due to complex MOF–polymer interactions and diverse underlying mechanisms for CO_2_ selectivity among different MOFs, selecting optimal MOF–polymer combinations for CO_2_ capture remains highly challenging.

As a starting point, it is important to consider the type of MOF and associated CO_2_ capture mechanism ideal for a given application. As described in section 2, engineering the MOF pore size is pivotal for enabling CO_2_ capture based on size exclusion. This property can be controlled by the choice of the organic linker, that is, via isoreticular expansion. This method involves selecting a linker with a different size but a similar molecular geometry of coordinating groups such that the resulting MOF retains the same network topology [104–106]. Recently, Gopalsamy et al. [106] computationally explored the substitution of the small oxalate linker in the parent MOF, CALF-20, by squarate, a slightly larger alternative linker. This modification expanded the pore-limiting diameter marginally from 2.8 to 2.9 Å, while maintaining the overall MOF topology. This isoreticular fine-tuning led to an increase in CO_2_/N_2_ selectivity from 180 to 500 in simulations of a 15/85 binary CO_2_/N_2_ mixture at 1 bar and 293 K.

Alternatively, incorporating pro-labile linkers that can be labilized and cleaved provides a way to expand or contract MOF pores post-synthetically in a controlled manner [107–108]. In addition, the choice of metal ion comprising the MOF nodes may also be altered to change the MOF pore size, though this may lead to changes in the selectivity due to specific metal interactions with CO_2_. Nevertheless, regulating the metal ions can enable pore size fine-tuning down to the sub-angstrom level [105,109–110]. For example, Shekhah and coworkers [110] synthesized SIFSIX-3-Cu, an isostructural variant of SIFSIX-3-Zn (3.84 Å), by employing CuSiF_6_ precursor instead of ZnSiF_6_ during synthesis. Substituting the larger Zn^2+^ ions with the smaller Cu^2+^ ions reduced the pore diameter to 3.50 Å and improved CO_2_/N_2_ selectivity in breakthrough experiments. It is important to note that various factors in the synthesis of MOF particles, such as the specific synthetic route, solvents, structure-directing agents, and precursors, can impact the final structure and yield of the synthesized MOFs. For more details on these effects, the interested reader is referred to the thorough review of MOF synthesis by Stock and Biswas [111].

In addition to MOF pore size tuning, the gas solubility and diffusivity within the polymer matrix must be optimized for the specific CO_2_ mixed gas separation application for optimal CO_2_ capture [112]. Particularly, the free volume within the polymer matrix and the polymer composition determine the gas diffusivity and solubility through the polymer matrix of the MMM [27]. Moreover, the MOF and polymer must be sufficiently compatible to avoid the formation of defects due to poor MOF–polymer matrix interfaces [90]. Notably, in addition to the benefit of providing LBSs for CO_2_ adsorption described in section 2, amino functionalization of MOFs before MMM preparation can enhance MOF–polymer compatibility by enabling the formation of hydrogen bonds between the MOF and the hydrogen bond donors in the polymer matrix [33].

#### 3.5 Importance of interfacial morphology

**Figure 6:**
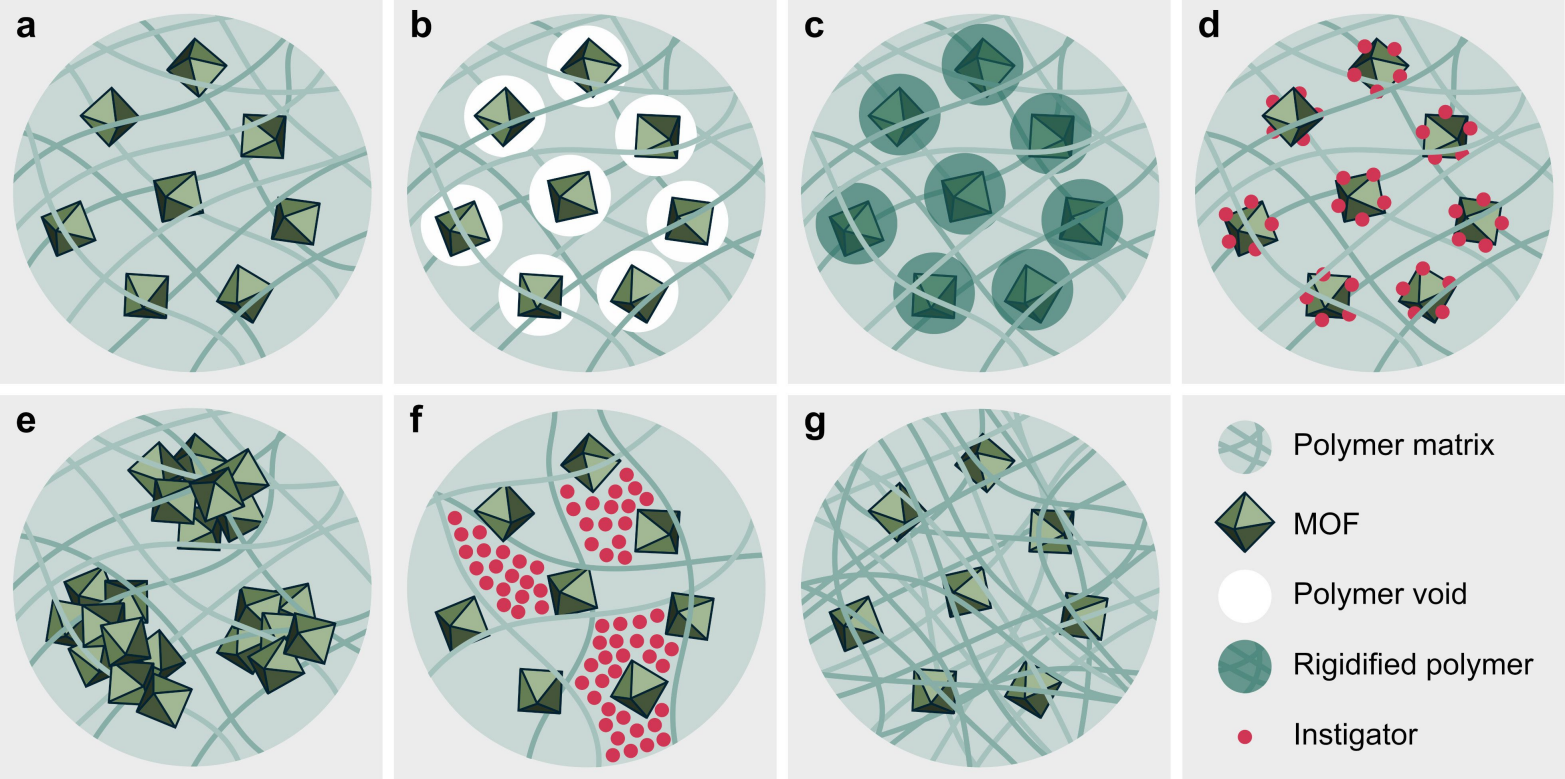
Illustration of different MOF–polymer matrix interfaces. (a) An ideal MOF–polymer matrix interface. (b) Poor adhesion between the MOF and the polymer leads to polymer voids around the MOF. (c) Rigid polymer immobilizing the MOF due to poor compatibility. (d) Instigating particles block the pores of the MOF, decreasing the permeability and changing the selectivity of the MMM. (e) Stochastically aggregated MOF particles within the polymer matrix. (f) Plasticization of the polymer matrix due to swelling from the infiltration of instigating particles. (g) Plasticization of the polymer matrix due to polymer chain equilibration.

An optimal MOF–polymer matrix interface, schematically illustrated in Figure 6a, is crucial for the gas separation performance of MOF-based MMMs, as it helps overcome defect formation and control interface morphology. There are five types of common defects for MOF-based MMMs, namely (1) voids around the MOF, Figure 6b, (2) rigidified polymer around the MOF, Figure 6c, (3) instigating particles blocking the MOF pores, Figure 6d, (4) aggregated MOF particles, Figure 6e, and (5) plasticization of the polymer chains, Figure 6f,g.

**Figure 7:**
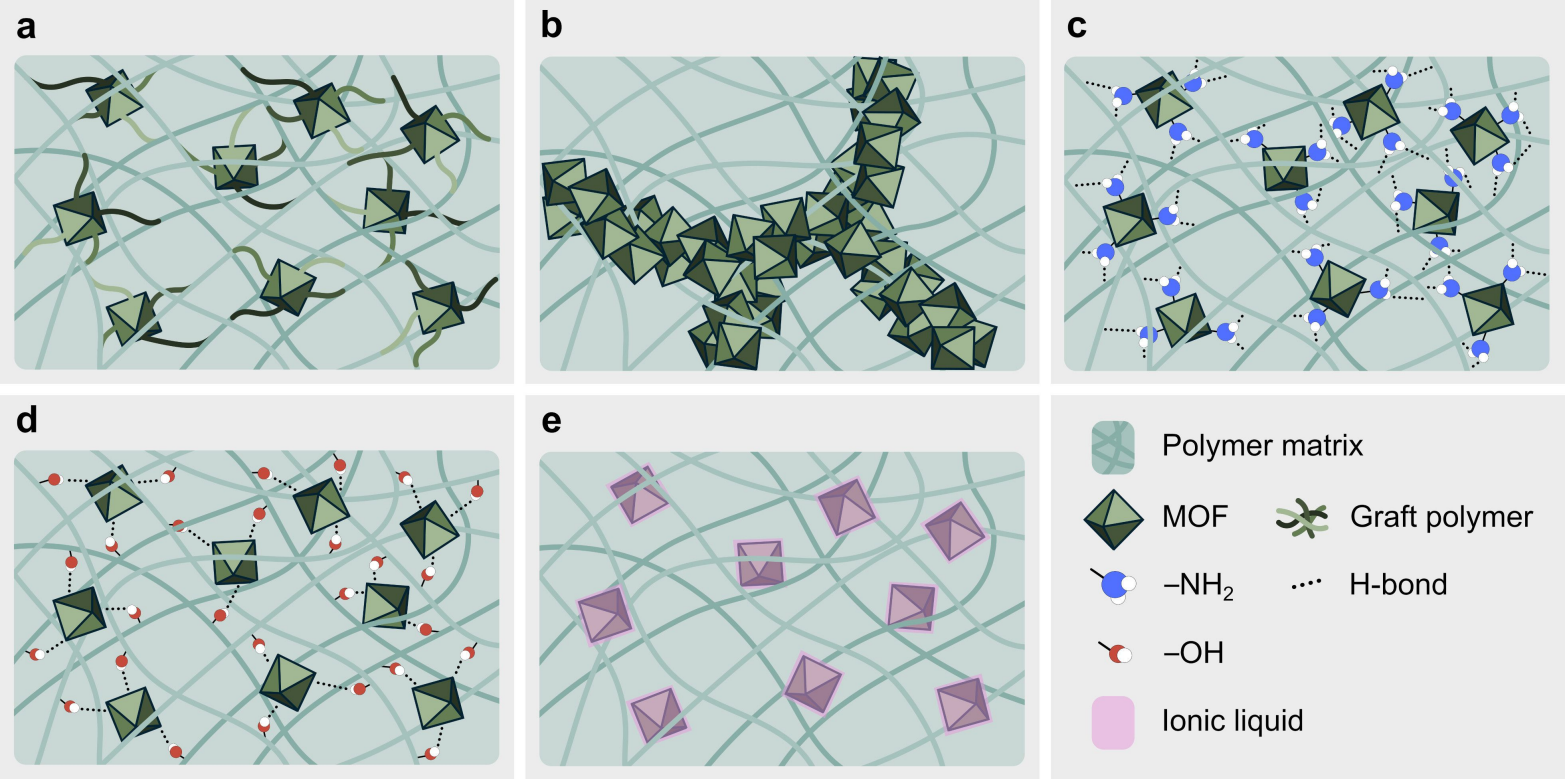
Illustration of different solutions to enhance MOF–polymer interfacial compatibility. (a) Polymer-grafted MOFs. (b) Percolation pathways through controlled MOF aggregation. (c) Amine-functionalized MOFs. (d) Hydroxy end group-functionalized polymer. (e) Ionic liquid-coated MOFs.

**3.5.1 Polymer voids.** Polymer voids, shown in Figure 6b, can drastically alter the selectivity and permeability of the MOF-based MMMs. For larger voids, the penetration of different molecules is no longer determined by the selectivity of the MOF, but rather by the size of the void. The voids arise from poor adhesion between the MOF and the polymer, emphasizing the importance of utilizing compatible components for MMMs [76,78,87,89,92,113]. Katayama et al. [113] found that a MMM with 50 wt % allyl-functionalized UiO-66 in polydimethylsiloxane (PDMS) exhibited macroscopic voids. The MMM had a CO_2_/N_2_ selectivity of just 1.0 ± 0.2 and a staggering CO_2_ permeability of 20115 ± 9275 Barrer. An equivalent MMM with an ideal MOF–polymer matrix interface, that is, after covalently grafting the UiO-66-PDMS interface, illustrated in Figure 7a, shows an increased selectivity of 10.0 ± 1.0, and the permeability was reduced to 4573 ± 727 Barrer.

**3.5.2 Rigidified polymer.** Rigidified polymer surrounding the MOF in the polymer matrix, illustrated in Figure 6c, can decrease selectivity and permeability. However, some degree of polymer rigidification can enhance CO_2_ separation performance by restricting polymer flexibility, which might otherwise reduce selectivity and cause unspecific permeation increases [114]. Additionally, a sufficiently rigid polymer matrix can help reduce plasticization effects [87,115]. Nevertheless, if rigidification is severe and localized to the polymer surrounding the MOFs, it can greatly decrease MMM permeability as CO_2_ molecules become restricted in their MOF diffusion [78,114,116]. In a 2004 publication by Moore et al. [117], it was demonstrated that existing MMM models consistently overestimated gas permeability in molecular sieve-integrated membranes, a discrepancy attributed to reduced polymer chain mobility at the polymer–sieve interface region, as evidenced by independent observations of increased glass transition temperature (*T*_g_) and suppressed permeabilities in the presence of fillers. A more recent example is the work by Ding et al. [118], which found that the preparation of PEO/ZIF-8 did not enhance the permselective performance compared to the pristine PEO rubber membrane due to significant rigidification effects and partial pore blockage. This rigidified polymer may also alter the effective MOF apertures, affecting MMM selectivity unpredictably [78,87,115,119]. The rigidified polymer defect typically forms during the solvent removal step, see Figure 4, where rapid solvent removal quenches the MOF-surrounding polymer [78]. In some cases, high MOF–polymer affinity can also cause local rigidification, reducing permeability greatly but possibly increasing selectivity [90]. Consequently, improving and controlling the MOF–polymer interface and fabricating the MMMs with slow solvent removal may reduce the rigidified polymer formation.

The overall rigidity of the polymer also has a large impact on the MOF–polymer interface. Rubbery polymers tend to reduce the propensity for interface defects, as the polymer chain mobility enables better confinement to the MOF surface [114,116]. However, as stated, the more flexible the polymer, the less selective, yet more permeable, it becomes. In contrast, glassy polymers, characterized by low chain mobility and increased rigidity, have the benefit of restricting the movement of gas molecules, but at the cost of reduced permeability and MOF compatibility [114,116,120]. Consequently, polymer rigidity is an important balance for any MOF-based MMM for optimal CO_2_ separation efficiency.

**3.5.3 Instigating particles.** Instigating particles hindering normal gas diffusion through the MOF, shown in Figure 6d, also affect CO_2_ permeability and mixed gas selectivity of the MMM, albeit to a lesser extent. These defects decrease permeability since they obstruct diffusion paths through the membrane. They also reduce selectivity since blocked MOF pores have altered effective apertures compared to the pristine MOF. The size of the instigator and its affinity to the MOF determine the extent of this effect [78,87,113]. Katayama et al. [113] also found that a blocked MOF interface resulted in a CO_2_ permeability decrease of nearly 50% and a CO_2_/N_2_ selectivity decrease of about 12% compared to an ideal MOF interface in the polymer matrix. Such defects typically arise during the preparation of the MOF-based MMM. Although prolonged gas exposure can cause fouling of membrane matrix and MOF pores, this is primarily observed in solution-based filtration applications [72,78,87–88]. Note that these defects may change the MMM selectivity favorably in some cases. In this way, the instigator may act as a tunable feature of the MMM that can be introduced before casting the MMM suspension [121].

For more information on polymer void, rigidified polymer, and instigating particle defects in a more general context, the reader is referred to the comprehensive research article by Moore and Koros [78].

**3.5.4 MOF aggregates.** Stochastic aggregation of MOF particles within the polymer matrix, as schematized in Figure 6e, can cause and facilitate other defects, such as polymer voids and rigidification. These aggregates may also introduce void space within the MOF clusters, resulting in additional selectivity loss [119]. The MOF aggregation propensity within the MMM tends to increase with higher MOF loading since MOF–MOF interactions are usually more favorable than MOF–polymer interactions. This makes it increasingly difficult to keep MOFs well-dispersed throughout the precursor slurry for higher MOF loadings [80,122]. Aggregation also tends to occur during the solvent removal step in the solvent casting method, further complicating MMM fabrication [122].

The tendency to form aggregates in MOF-based MMMs depends on the specific MOF and polymer used, their interactions, and the fabrication process. A recent study by Li et al. [123] revealed that controlled aggregation in flat sheet MMMs from polymer blends of two immiscible polyimides and UiO-66-NH_2_ can significantly enhance CO_2_ separation performance for CO_2_/CH_4_ and CO_2_/N_2_ binary mixtures. Specifically, they demonstrated that fine-tuning parameters like MOF loading, polymer ratio and composition, and solvent evaporation rate could mitigate the typical defects associated with stochastic MOF aggregation, even creating percolation pathways at MOF loadings of 19 wt %, Figure 7b. These percolation pathways acted as CO_2_-specific channels within the MMM, forming an interconnected network of MOF particles. This structural property increased CO_2_ permeability without loss of selectivity compared to regular MOF-based MMMs with uncontrolled aggregation.

Some MMM systems synthesized through standard solution casting, as illustrated in Figure 4, can accommodate MOF loadings of 30 wt % without notable aggregation effects [92], whereas others experience strong aggregation beyond, for example, 16 wt % MOF loading [91]. A well-established and simple method to reduce aggregation at higher MOF loadings is to prime the MOF slurry with a small amount of polymer. More advanced techniques include MOF functionalization and polymer grafting onto the MOF to significantly improve the MOF–polymer affinity, allowing MOF loadings up to 50 wt % without aggregation [113].

**3.5.5 Polymer plasticization.** Finally, plasticization is a common effect in MMMs in general and can occur as a type of physical aging of the MMM [119]. This effect typically impacts the entire polymer matrix of the MMM. It may result from the infiltration of many instigating particles, causing swelling of the porous sites in the polymer matrix, as shown in Figure 6f. Plasticization can also arise because of polymer chain equilibration, where, over time, the polymer chains rearrange toward thermodynamic equilibrium. This results in the expulsion of free volume from the polymer matrix, which reduces the pore size in the polymer matrix, as seen in Figure 6g. Both types of plasticization reduce CO_2_ selectivity. However, swelling plasticization increases permeability, while equilibration plasticization decreases it [119,124–125]. MOFs within the membrane matrix mitigate this issue compared to pristine organic polymer membranes. Nevertheless, dedicated strategies such as polymer cross-linking [126] or MOF geometry tuning [127] are needed to address this issue properly. Furthermore, the study by Maleh and Raisi found that in situ MOF-based MMM synthesis resulted in negligible plasticization compared to ex situ MMM fabrication [91], suggesting that MOF distribution and MOF–polymer matrix interface compatibility are crucial for reducing plasticization.

**3.5.6 Reducing defect propensity.** Besides the techniques already discussed, the formation of defects in MOF-based MMMs can be significantly reduced by optimizing the MOF–polymer interface through MOF or polymer functionalization, Figure 7c,d, reducing filler sizes, in situ MOF growth, or applying annealing treatments. Interfacial adhesion can also be improved using ionic liquids (Figure 7e).

MOF functionalization is commonly employed in MMM research. For example, in the study by Katayama et al. [113] mentioned earlier, the challenges related to the MOF–polymer interface were addressed through enhanced MOF–polymer interactions in a PDMS matrix by grafting allyl-substituted UiO-66 with a PDMS corona. Upon MMM preparation, the MOF-grafted PDMS corona was covalently bound to the PDMS polymer matrix, resulting in defect-free MMM as illustrated in Figure 7a. In another study, Khosravi et al. [33] functionalized HKUST-1 with amines to improve adhesion with a Pebax^®^ 1657 polymer matrix through hydrogen bonding, illustrated in Figure 7c. Differential scanning calorimetry showed an increase in *T*_g_ of the MMM, indicating reduced polymer chain flexibility. The effect was more pronounced in NH_2_-HKUST-1/Pebax^®^, suggesting the formation of hydrogen bonds between the amine-functionalized MOF surface and polymer chains. Alternatively, optimizing the MOF–polymer interface can be achieved through polymer functionalization, Figure 7d. For instance, in a study by Carja et al. [128], PIM-1 was functionalized with amidoxime groups to induce superior adhesion to UiO-66 in flat sheet MMMs, drastically reducing defect formation as predicted by molecular simulations and confirmed by field-emission scanning electron microscopy and high-resolution transmission electron microscopy (HRTEM).

Utilizing smaller MOF filler sizes is a straightforward approach to improve filler dispersion. The contact area with the polymer matrix increases when the size of MOF particles is decreased, promoting adhesion, and reducing interfacial defects [80]. Larger MOF particles will increase the permeability of all gases, which may decrease specific gas selectivity [94]. However, smaller MOF particle sizes can also increase aggregation tendencies [87]. Consequently, optimizing the MOF particle size remains a case of trial and error on a case-by-case basis.

An entirely different approach to improving the MOF–polymer interface is shown in the study by Maleh and Raisi [91], where ZIF-8 MOFs were grown in situ in a Pebax^®^ 2533 polymer matrix. The authors prepared a polymer solution in ethanol containing well-dispersed zinc nitrate hexahydrate and another polymer solution with 2-methylimidazole. Nucleation and MOF growth occurred in situ within the polymer matrix upon combining the two precursor solutions. This approach resulted in nearly perfect MOF–polymer interfaces in an 8 wt % ZIF-8 MMM. For evaluating the gas separation performance, the authors prepared an ex situ MMM control through plain solvent casting, as illustrated in Figure 4c, using the priming technique. Upon comparison, they found that CO_2_/CH_4_ and CO_2_/N_2_ selectivity were drastically improved for the in situ MMM, albeit with slightly lower CO_2_ permeability.

The interaction between polymer and fillers has also been enhanced through annealing treatments. Although annealing of MOFs can cause partial decomposition of the structural framework by imposing local defects, such treatments have been shown to increase CO_2_ uptake capacity in MOFs [129] and improve CO_2_ capture performance of MOF-based MMM [130]. In a study by Lai et al. [130], ZIF-8 was annealed at 300 °C for 1 h before being mixed with a poly(styrene-*co*-butadiene) solution during MMM fabrication. For filler loadings of 10 to 30 wt %, annealing led to an increase in CO_2_ permeability and CO_2_/N_2_ selectivity due to better MOF–polymer compatibility and promotion of CO_2_ affinity caused by a defect-driven exposure of unpaired –N bonds in the MOF framework. Annealing treatments conducted after membrane casting have also been shown to improve CO_2_/CH_4_ separation of a ZIF-8/Matrimid^®^ MMM [131].

**Figure 8:**
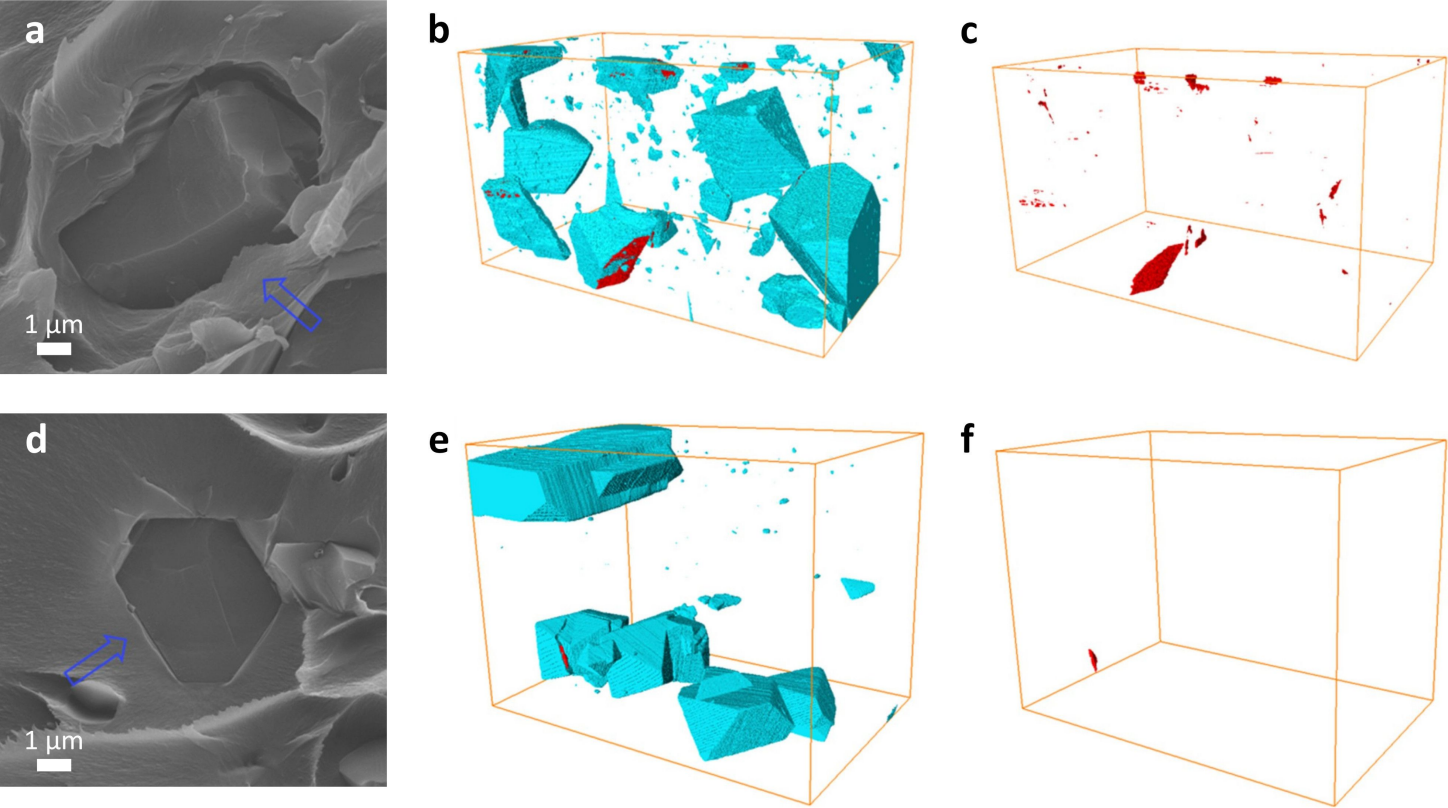
Cross-sectional SEM images of (a) HKUST-1 MMM and (d) HKUST-IL MMM and surface-rendered view of segmented FIB-SEM tomograms of (b, e) fillers and voids and (c, f) voids for (b, c; box size 35.7 µm × 26.5 µm × 23.3 µm) HKUST-1 MMM and (e, f; box size 40.7 µm × 24.0 µm × 23.0 µm) HKUST-IL MMM. Blue arrows point out MOF–polymer interfaces. Filler appears in cyan blue, and voids in red [132]. Adapted with permission from [132], Copyright 2016 American Chemical Society. This content is not subject to CC BY 4.0.

Furthermore, specific additives that enhance the adhesion between MOFs and the polymer matrix can be used to improve the MOF–polymer interface. For example, Lin et al. [132] utilized [Emim][Tf_2_N], an ionic liquid (IL), as an interfacial binder between micrometer-sized HKUST-1 and 6FDA-Durene polymer, see Figure 8e. The application of the IL demonstrated effective adhesion between MOF–IL and IL–polymer interfaces. This resulted in a noticeable reduction of interfacial voids within the membrane, thereby facilitating a significant increment in CO_2_ selectivity [132]. The use of ILs (e.g., [P(3)Him][Tf_2_N], [Bmim][Tf_2_N], and [Dmim][Cl]) in the preparation of MOF-based MMMs has been demonstrated in numerous other studies on CO_2_ separation [97,133–138].

Note that all the previous considerations are generally true for MOF-based MMMs for CO_2_ capture purposes.

#### 3.6 Structural characterization of MOF-based MMMs

Material characterization techniques are pivotal for the assessment of novel MOF-based MMMs structures. Electron microscopy enables direct imaging of a sample with up to sub-nanometer resolution [139]. Scanning electron microscopy (SEM) is a popular and straightforward method to obtain images of MOF-based MMMs [113,118,122,124–125,128,131–132]. Often, the membrane is broken apart to enable a cross-sectional view of the MOF-based MMM, which may effortlessly reveal interfacial defects as in Figure 8 [132]. In addition to SEM, TEM and HRTEM are often used to obtain information about MOF distribution within the MMM [122,125,128]. However, TEM analysis of MOF-based MMMs usually involves diluting the MOF–polymer precursor slurry in a volatile solvent and dispersing it on a conductive metal grid. The sample is then dried in air or under vacuum [122,125]. Consequently, the ultrathin membrane formed may not fully represent the structure of the fabricated MOF-based MMMs.

While electron microscopy offers a highly detailed qualitative analysis of specific regions within the membrane, methods such as Fourier-transform infrared spectroscopy (FTIR), nuclear magnetic resonance (NMR) spectroscopy, and X-ray diffraction (XRD) provide information about the bulk MOF-based MMM [140–142]. FTIR yields information about the functional groups within the sample [140], and will differ between the pristine MOF, polymer membrane, and MOF-based MMM [118,121–122,124,128,131]. In this way, it is possible to compare how various modifications to the MOF-based MMM system change the functional groups within the final membrane [122,125,128]. Similar to FTIR, NMR provides insights into the chemical nature of the membrane but offers more detailed information about its chemical structure [141]. XRD supplements the material analysis with crystallographic data [142]. As MOFs are crystalline, crystallographic spectra of MOFs, polymer membranes, and MOF-based MMMs will differ notably, which in turn may indicate the success of MOF integration in the polymer membrane [113,118,122,131]. Alterations to the MOF-based MMM system that perturb the crystallinity in the membrane will be discernable through XRD [121,129].

Energy-dispersive X-ray spectroscopy (EDX), and X-ray photoelectron spectroscopy (XPS) are commonly used to supplement the chemical analysis of MOF-based MMMs [113,137,143]. EDX can provide information on the chemical composition of the outermost 1–3 µm of a material [144–145]. A visual and spectral representation of the chemical composition of a MOF-based MMM cross section may help identify the presence and type of defects within the membrane and simply verify the successful integration of MOFs in the polymeric membrane [113,137,143,146–147]. XPS is commonly used for obtaining spectra of the chemical composition of the outermost 10 nm of a surface. Specifically, XPS provides a spectral representation of binding energies on the surface, which can be referenced to specific chemical states [148]. In MOF-based MMMs, XPS can help elucidate unique chemical coordination within the membrane [143].

Physical properties such as the surface area and pore size distribution are commonly determined through Brunauer–Emmett–Teller (BET) analysis [121,124–125,129–131], where the BET theory is applied to adsorption isotherms measured for the membrane with an inert gas such as nitrogen [149]. The method remains a standard within MOF-based MMMs for determining surface area, pore size, and pore distribution.

A less commonly used but highly potent technique is positron annihilation lifetime spectroscopy (PALS). The PALS method offers a unique way of measuring the size and distribution of free volume within a membrane [122]. PALS is based on variances in positron lifetimes due to an uneven electron density across a sample, arising from the free volume distribution [150]. Utilization of PALS for MOF-based MMMs may reveal whether a change in free volume can be attributed to void defects, alterations in the amount of integrated MOF in the membrane, or changes in the polymer packing [122,131].

The reader is referred to the following literature for well-written introductory material on the mentioned characterization methods: SEM by Vernon-Parry [145], TEM by Franken et al. [151], FTIR by Mohamed et al. [140], NMR by Kwan et al. [141], XRD by Bunaciu et al. [142], EDX by Hodoroada [144], XPS by Stevie and Donley [148], the BET method by Sinha et al. [149], and PALS by Jean and coworkers [150].

### 4 Performance, permeability, and selectivity trade-off

A key issue with regular organic polymer membranes used for CO_2_ separation is that although they display some degree of selectivity and permeability, one property can only be tuned at the expense of the other [5]. This phenomenon is commonly referred to as Robeson’s upper bound, which describes the performance boundary of the membrane selectivity in relation to its permeability and vice versa. Conversely, as the selective properties of the membrane are improved, the permeability decreases within this upper bound [13].

#### 4.1 The case of HKUST-1 based MMMs for CO_2_ separation

**Figure 9:**
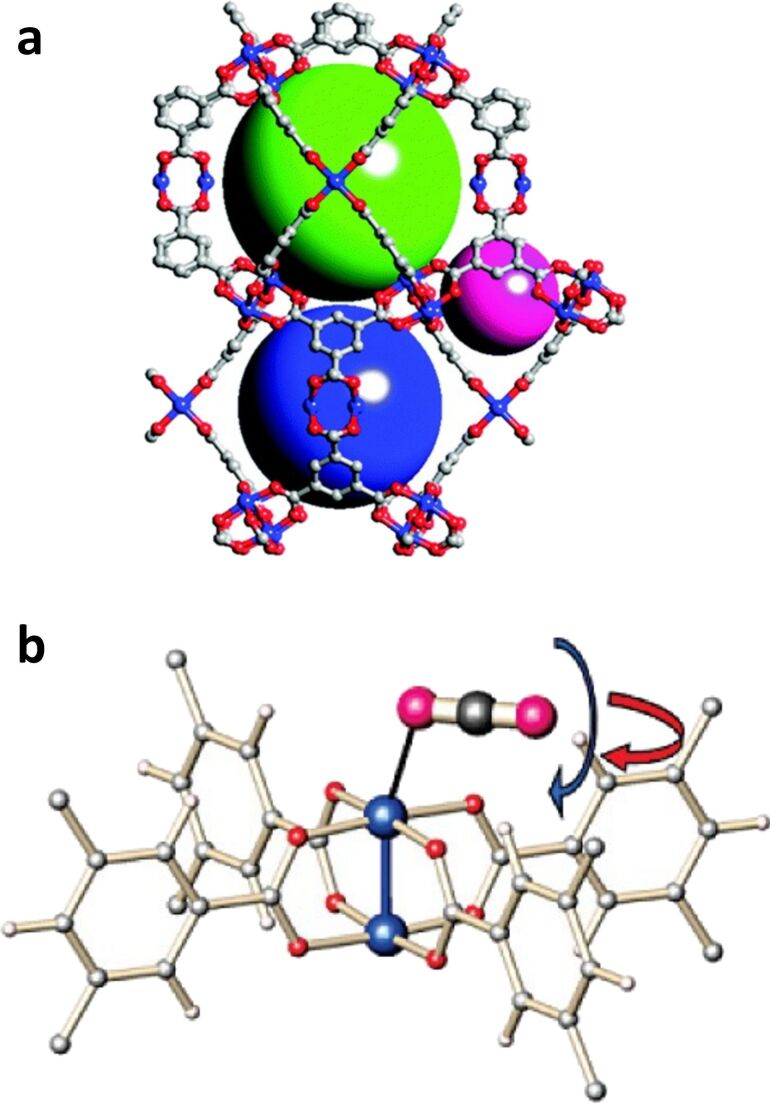
(a) HKUST-1 structure with cavities of 14 Å (green sphere), 11 Å (blue sphere), and 5 Å (purple sphere) pore diameter. Used with permission of The Royal Society of Chemistry, from [163] (“Methane storage in metal-organic frameworks”, by Y. He et al., Chem. Soc. Rev., vol. 43, issue 16, © 2014); permission conveyed through Copyright Clearance Center, Inc. This content is not subject to CC BY 4.0. (b) CO_2_ adsorption at the open Cu(II) sites [63]. The color scheme of framework atoms: Cu, blue; C, gray; O, red; and H, white. The color scheme of the CO_2_ atoms: C, gray; O, purple. Adapted with permission from [63], Copyright 2010 American Chemical Society. This content is not subject to CC BY 4.0.

A recent review by Rangaraj et al. [5] thoroughly examined the CO_2_ separation performance of MMMs employing common MOF fillers and composites. However, the review did not highlight HKUST-1 despite its outstanding CO_2_ capture properties [152], and its employment in the fabrication of MMMs [33,132,138,146,153–158]. Characterized by the chemical composition Cu_3_(BTC)_2_, HKUST-1 features Cu^2+^ coordinated by 1,3,5-benzenetricarboxylate (BTC) linkers. The structure of HKUST-1 comprises face-centered cubic crystals with Cu_2_(OAc)_4_ paddlewheel-type secondary building units [159], forming a 3D lattice with square-shaped pores [160]. These pores exhibit diameters of 14, 11, and 5 Å. Given its rather large pore size compared to the kinetic diameter of CO_2_, it is widely acknowledged that the primary adsorption mechanism of CO_2_ onto pristine HKUST-1 involves coordination to the OMSs, driven by the strong electrostatic interaction between Cu^2+^ and the quadruple moment of CO_2_ [63,161–162], illustrated in Figure 9.

Most HKUST-1-based MMMs are prepared by solution casting [146,154], but examples of phase inversion fabrications are also evident in the literature [156]. As highlighted in section 3.5, excessive MOF loadings tend to induce significant stress on the MMM due to chain rigidity and particle aggregation [153]. Past a specific filler loading threshold, MMMs often contain more defects, making them brittle and prone to breakage after casting or in operation [146]. Table 1 outlines a range of studies exploring MMMs that employ HKUST-1-based fillers for CO_2_ separation. The best-performing MMM in each study is evaluated based on key performance metrics including CO_2_ permeability (measured in Barrer, where 1 Barrer = 10^–10^ cm^3^(STP)·cm·cm^–2^·s^–1^·cmHg^–1^) and CO_2_ selectivity over CH_4_ and N_2_.

**Table 1:** Performance of HKUST-1-based MMMs for CO_2_ capture.^a^

HKUST-1 filler	Polymer matrix	*P* (bar)	*T* (°C)	Permeability (Barrer)	Selectivity CO_2_/CH_4_	Selectivity CO_2_/N_2_	Ref.

HKUST-1	PDMS	—	—	2 900	3.6	8.9	[153]
HKUST-1	6FDA-*m*PD	2	35	28.3 ± 0.4	89 ± 4	ND	[146]
HKUST-1	6FDA-ODA	150	35	21.8	51.2	ND	[154]
HKUST-1	Matrimid^®^ PI	10	35	17.5^b^, 19.5^c^ GPU	23.0	23.5	[156]
HKUST-1	6FDA-DAM	1.03	35	2 360 ± 80	14.9 ± 0.7	14.6 ± 0.7	[125]
sub-HKUST-1	[Emim][Ac]-chitosan	2	50	4 754 ± 1 388	ND	19.3	[138]
Gly@HKUST-1	Pebax^®^ 1657	2	25	175	29	ND	[143]
NH_2_-HKUST-1	Pebax^®^ PEBA	3	30	163.7	26.2	ND	[33]
sub-NH_2_-HKUST-1	Pebax^®^ PEBA	1	25	108.5	29.05	66.27	[157]
HKUST-1/[Emim][OAc]	Pebax^®^ 1657	1	35	335 ± 7	ND	176.3 ± 4	[164]
HKUST-1/[Emim][Tf_2_N]	6FDA-Durene	2.03	25	145 ± 4.7	19.6 ± 1.5	17.9 ± 1.2	[132]
HKUST-1/[Dmim][Cl]	Polysulfone	4	30	30.08 GPU	24.36	25.72	[147]
HKUST-1/[Bmim][Tf_2_N]	Polysulfone	4	30	30.59 GPU	26.46	27.39	[147]
HKUST-1/GO	PVDF	5	25	3.316	66.32	ND	[155]
HKUST-1/S1C	Udel^®^ PSF	2.75	35	8.9^b^, 8.4^c^	22.4	38.0	[158]
HKUST-1 with interconnected branches	6FDA-DAM	1.03	35	2 480 ± 80	16.5 ± 0.8	16.3 ± 0.8	[125]

^a^Information about HKUST-1 loading, gas mixture composition, and experimental setup can be accessed in the source publications. Abbreviations: not determined (ND), silicate-1 (S1C), graphene oxide (GO), gas permeation unit (GPU; Barrer/µm(membrane thickness)); ^b^CO_2_/CH_4_ separation; ^c^CO_2_/N_2_ separation.

In the earliest reported case of an HKUST-1-based MMM, Car et al. [153] prepared a MMM using a rubbery PDMS polymer matrix and pristine HKUST-1 at a 10 wt % loading achieved through solution casting. This yielded a CO_2_ permeability of 2900 Barrer and CO_2_/CH_4_ and CO_2_/N_2_ selectivity of 3.6 and 8.9, respectively. Subsequent advancements in MMM design include fine-tuning the matrix with various polymers, resulting in materials with significantly higher CO_2_ selectivity, albeit at the expense of permeability [146,154,156]. For instance, Nuhnen et al. [146] achieved excellent results by preparing a MMM with a 24 wt % HKUST-1 loading in a 6FDA-*m*PD polyimide matrix, attaining α_CO2/CH4_ = 89 and a permeability of 28.3 Barrer. In other studies, researchers aim to optimize CO_2_ capture performance by tailoring filler geometry and size, functionalizing ligands, and incorporating composite fillers and ionic liquids during MMM fabrications [33,125,132,138,155,157–158].

#### 4.2 Overcoming the Robeson upper bound

**Figure 10:**
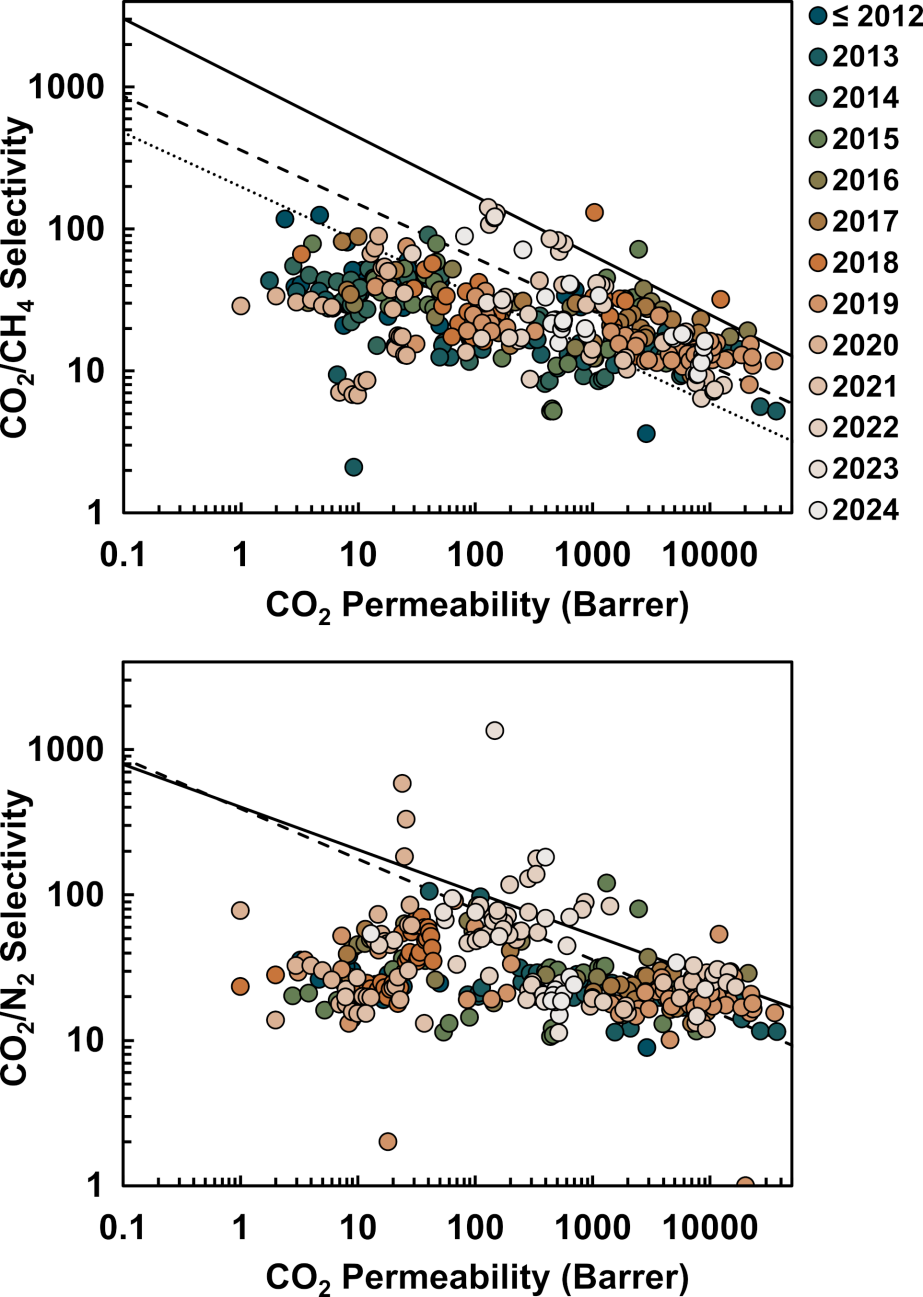
Selectivity–permeability plots of MOF-based MMMs for CO_2_ capture reported in the literature. Detailed data are available in Supporting Information File 1. Lines indicate the 1991 (dotted) and 2008 (dashed) Robeson upper bound, as well as the 2019 upper bound proposed by Comesaña-Gándara and coworkers [13–14].

Performance evaluation is paramount for advancing membrane-based gas separation technologies for CO_2_ capture. In this context, the permeability–selectivity trade-off benchmark, the Robeson upper bound, serves as an aspirational target for researchers aiming to design MMMs that approach or surpass this metric. Advancements in membrane technology driven by material innovation and computational modeling have led to the redefinition and rise of CO_2_/CH_4_ and CO_2_/N_2_ upper bounds over time, as illustrated in Figure 10. While a considerable portion of current published research on MOF-based MMMs trails behind the most recently defined upper bound, some manage to exceed it.

A versatile group of MOF-based MMMs are ‘smart MMMs’ based on flexible MOF fillers that adapt to environmental changes [76]. Xin et al. [21] recently developed a MMM with Co(AzDC) fillers in a Matrimid^®^ 5218 matrix. Co(AzDC) possesses a light-responsive azo group from the 4,4′-azobenzenedicarboxylic acid ligand that undergoes *cis*–*trans* isomerization by tuning UV light irradiation time or intensity. Under optimal conditions, a CO_2_ permeability of 156 Barrer and a CO_2_/N_2_ selectivity of 78 was reached for a 10/90 CO_2_/N_2_ feed mixture, thus overcoming the 2008 Robeson upper bound.

**Figure 11:**
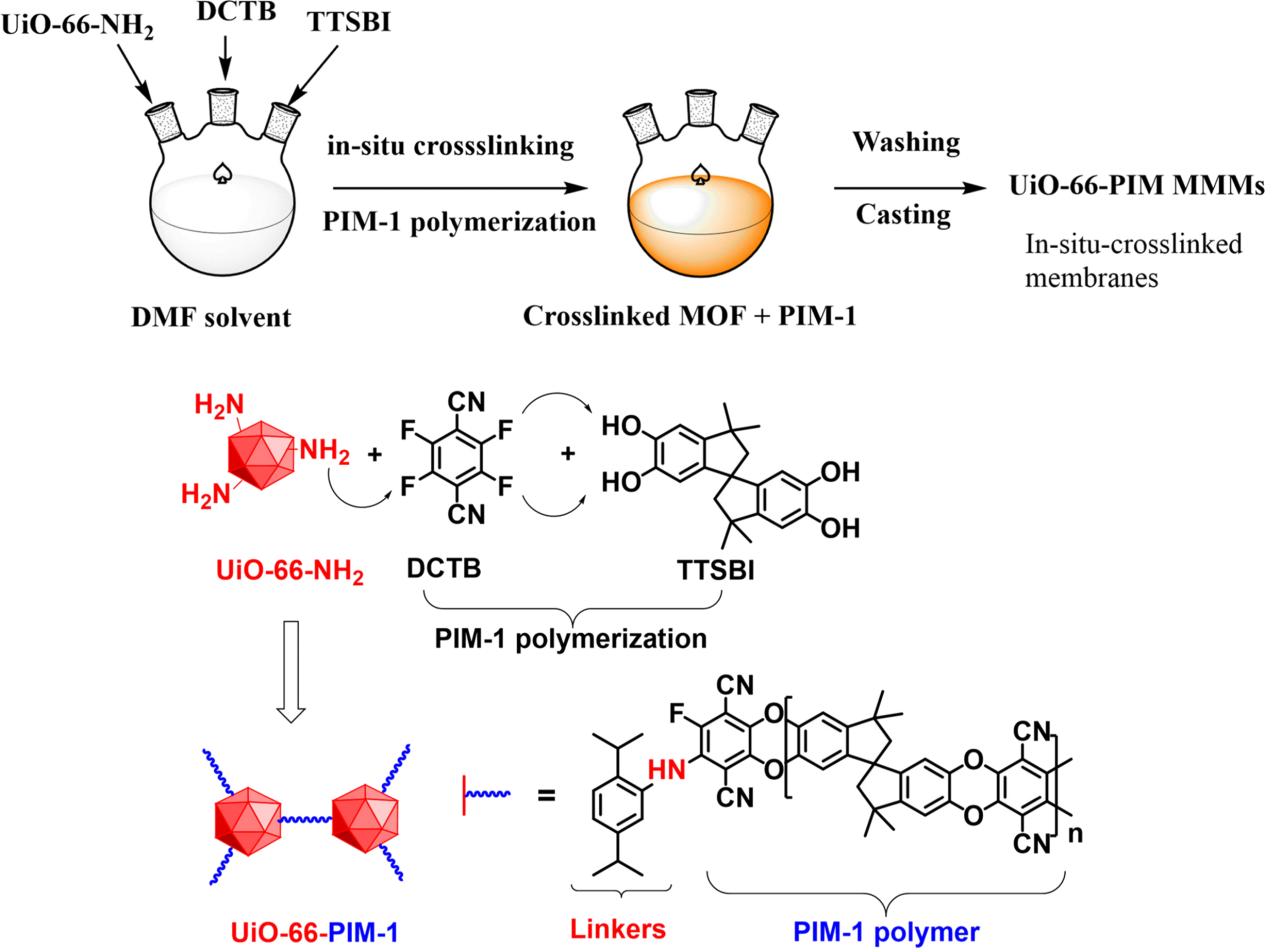
Schematic diagram of the UiO-66-PIM preparation method and cross-interface linking between UiO-66-NH_2_ and PIM-1 obtained during the in situ polymerization [165]. Reprinted from [165], Journal of Membrane Science, vol. 548, by N. Tien-Binh; D. Rodrigue; S. Kaliaguine, “In-situ cross interface linking of PIM-1 polymer and UiO-66-NH2 for outstanding gas separation and physical aging control”, pages 429-438, Copyright (2017), with permission from Elsevier. This content is not subject to CC BY 4.0.

In a 2018 study, Tien-Binh et al. [165] achieved remarkable results, surpassing the 2019 upper bounds of CO_2_/CH_4_ and CO_2_/N_2_ mixtures. Their work demonstrated a CO_2_ permeability of 12 498 Barrer, along with selectivities α_CO2/CH4_ = 31.9 and α_CO2/N2_ = 54.2, recorded at 25 °C and 2 bar for UiO-66-PIM at a 20 wt % MOF loading. As opposed to the traditional method of preparing MMMs by simply mixing presynthesized polymers and fillers before casting, this study introduced an innovative in situ polymerization approach detailed in Figure 11. Through this approach, a rigid polymer of intrinsic microporosity (PIM-1) and an amine-functionalized MOF (UiO-66-NH_2_) were chemically linked during polymerization, resulting in cross-linked MMMs exhibiting exceptional adhesion between the polymer and filler and providing the MMM with molecular-level homogeneity. This resulted in an interconnected micropore network throughout the MMMs, eliminating interface voids and facilitating rapid gas transport.

The work by Tien-Binh et al. [165], Figure 11, represents just one example of the promising development of MOF-based MMMs. Other achievements worth mentioning are innovative MMM fabrications such as a bottom-up synthesis of ZIF-8-integrated PIM-1 MMMs in situ, inspired by the symbiotic relationship between rhizobium bacteria and plant roots reported by He et al. [166], or a novel carbon nanotube–MOF complex, which integrates well with polyamide-based MMMs for superior gas permeation reported by Lin and coworkers [167]. Despite recent achievements that push the boundaries for MOF–polymer interfacial compatibility, MOF dispersion, and MOF loading, these improvements should be better reflected in performance metrics. Trends reported in Figure 10 indicate large performance variability, emphasizing the need for a more systematic and standardized approach to design and test new MMMs to surpass current benchmarks. In particular, CO_2_/CH_4_ separation has not shown improvement in recent years. Given the lack of a universal one-size-fits-all MOF-derived MMM for CO_2_ capture, targeted research to specific applications should be intensified. Prediction-based computational studies can play an important role in achieving these objectives.

### 5 Design and prediction through machine learning and computational modeling

Determining the optimal MMM composition for CO_2_ capture is a challenging task, as emphasized in section 3.4. The sheer number of available and potentially customizable MOFs, combined with the wide variety of polymers suitable for MMM design, makes evaluating all relevant combinations for MMM-based CO_2_ capture experimentally impractical. Machine learning (ML) and associated deep learning models leverage substantial data to model, test, and aid in interpreting large and complex systems and have driven considerable advancements in many areas of natural sciences [168–170]. Similarly, ML may hold significant potential for identifying optimal MOF–polymer combinations for CO_2_ capture.

Computational modeling (CM) in the form of molecular dynamics (MD), a statistical modeling technique, has become a well-established method for simulating molecular behavior in many different systems, such as water distribution and arrangement in hydrogels [171], interactions between muscle proteins and stabilizing additives [172], and the behavior of lithium polysulfides in lithium–sulfur batteries [173]. Recently, MD simulations have been explored in CO_2_ capture with MOF-based MMMs [174–177], showing promise in identifying MOF–polymer combinations for novel MOF-based MMMs for CO_2_ capture.

#### 5.1 Machine learning and molecular dynamics in the context of MOF-based MMMs

Fundamentally, ML aims to develop an algorithm that links certain data characteristics to particular outcomes [178]. In the context of CO_2_ capture with MOF-based MMMs, this involves relating properties such as composition, physical and chemical characteristics, and stability of the pristine MOF and polymer components, as well as their interactions, to the CO_2_ separation performance of the final MMM from a given gas feed mixture [179]. These algorithms are produced by training a ML model on data with known property–outcome relationships and fine-tuning the model predictions using validation data sets [178]. For a non-technical introduction to ML, the interested reader is referred to the review by Choi and coworkers [178]. In contrast, CM involves carefully considering physical, chemical, biological, and mathematical concepts to construct a model that accurately describes the complex dependence of a data set on various system parameters [180–181]. For a thorough discussion of different CM approaches for MOF-based MMMs, the reader is referred to the review by Keskin and Alsoy Altinkaya [114].

MD is a subset of CM that attempts to fit the time-dependent behavior of a system to Newton’s equations of motion. Through this approach, MD offers time-averaged parameters within the constructed model, enabling the prediction of real experimental behavior [182]. In evaluating the gas separation performance of MOF-based MMMs for MOF–polymer combinations, MD is employed to model the movement of gas molecules through the various types and sizes of MMM pores. Mostly, the grand canonical Monte Carlo (GCMC) technique is used [114]. GCMC simulations generate many stochastic molecular configurations and construct a probability distribution that reveals average system properties, such as gas adsorption. Specifically, GCMC simulations are restricted to a fixed volume, temperature, and chemical potential, in contrast to regular Monte Carlo (MC) simulations [183]. For both regular MC and GCMC simulations, force fields with well-defined interatomic potentials are utilized [114,183].

Overall, two GCMC-based MD approaches are used to model MOF–polymer pairs for MOF-based MMMs. Either (1) MOF gas permeability is simulated and combined with experimental polymer gas permeability data for model construction, or (2) both MOF and polymer gas permeability are simulated for model construction. These simulations are conducted at atomic-level detail assuming perfect MOF–polymer adhesion and no MOF defects. The first approach inherently lacks atomic-level detail for gas diffusion through the polymeric pores, potentially leading to deviations compared to real data. In contrast, the second approach requires extensive computational demands but provides higher-quality simulations [114].

While CM involves carefully considering the system properties to construct an accurate model, ML employs algorithms trained on extensive datasets to generate predictive models for testing [178,180]. Both approaches can utilize extensive databases on MOFs, for example, the Cambridge Structural Database MOF Subset [184], ARC-MOF [185], and CoRE MOF [186], and polymer membranes, for example, the Polymer Gas Separation Membrane Database [187], to rapidly obtain data for model development [29,188]. This data can be curated to facilitate a streamlined selection of MOFs and polymers for further study. Upon CO_2_ capture based on size exclusion for instance, it is typical within CM to first determine the structural properties of the MOFs in the utilized data set to exclude MOFs with pore sizes smaller than the kinetic diameter of CO_2_ at 3.3 Å, before proceeding with simulating adsorption and diffusion of gas molecules [29].

#### 5.2 Predicting MOF-based MMM CO_2_ capture performance

While research in this area is still in its early stages, some efforts have been made to computationally screen MOFs and polymers using ML to identify promising MMM designs for CO_2_ capture. A study by Guan et al. [179] demonstrates the promising use of ML to predict the CO_2_ separation performance in MOF-based MMMs. The authors employed a random forest model, which utilizes decision trees, a specific type of ML algorithm that partitions data according to set criteria until a certain stop condition is met. While decision tree algorithms tend to overfit provided data, the random forest model mitigates this by using only a random subset of the obtained results from many different fits [189]. Guan and coworkers [179] trained their model on 648 data sets consisting of 36 MOFs and 41 polymers, considering the intrinsic MOF properties, polymer matrix properties, MMM characteristics, and experimental conditions. Their trained model identified that an optimal MOF structure for high CO_2_ selectivity and permeability during CO_2_/CH_4_ and CO_2_/N_2_ separation should have a pore size larger than 10 Å and a BET surface area of approximately 800 m^2^·g^−1^. The researchers validated their predictive model by synthesizing MMMs with Cu-CAT-1 and Cu-THQ fillers and testing their CO_2_ separation performance, observing good alignment between prediction and experimental results.

Another useful example is the recent study by Yao et al. [190], where the group constructed an artificial neural network (ANN) based on 291 different MOF-based MMMs for CO_2_/N_2_ separation. They then optimized the ANN using a genetic algorithm (GA) to predict optimal MOF–polymer pairings for CO_2_ capture. ANNs function analogously to biological neural networks, dividing tasks into multiple information streams and processing them through interconnected nodes, similar to neurons. These nodes handle information differently based on varying input–output weights and are configured to optimize information transmission. The entire node network is regulated and tuned by learning rules that adapt the algorithm to the data [191]. GAs mimic natural evolution and selection by introducing ‘mutations’ in a solution space, then ‘breeding’ optimal solutions, and proceeding only with the most optimal solutions based on set criteria [192]. For their ANN, Yao et al. linked MOF and polymer properties and operating conditions to CO_2_ separation performance for mixed gas feeds. The authors then applied the GA to optimize the important features of CO_2_ mixed gas separation performance, using a mutation probability of 1% over 100 generations. This approach yielded an algorithm with determination coefficients of 0.97 for the permeability fit and 0.90 for the selectivity fit. Finally, they used the Shapley additive explanation method to examine their model [190]. The Shapley method evaluates ML models by scoring the importance of different system variables in the system variability [193]. This analysis identified the MOF type, filler size, pore size, and surface area to be among the most important factors for CO_2_ permeability, along with the chemical and topological properties of the polymer. For CO_2_/N_2_ selectivity, the same MOF properties, except filler size, were found to be important, in addition to the polymer type and the operational temperature of the MMM.

Numerous studies have leveraged CM to evaluate CO_2_ mixed gas separation performance. An example is the study by Altintas and Keskin [174], which employed extensive GCMC and MD simulations to assess CO_2_/CH_4_ gas separation performance for 3794 defect-free and perfect single-crystal MOFs, from the Cambridge Structural Database MOF Subset [184]. They identified the eight most promising MOF candidates for CO_2_/CH_4_ gas separation (CO_2_ permeability > 10^6^ Barrer, α_CO2/CH4_ > 80) and studied them with eight different polymers, yielding 64 unique MOF-based MMMs. The gas separation performance of these MMMs was evaluated through the theoretical Maxwell permeation model [194]. In most cases, incorporating MOFs in the polymer matrixes was predicted to improve CO_2_ permeability without significantly affecting CO_2_ selectivity. In just a few instances, both permeability and selectivity were significantly enhanced. NURVAZ/PIM-1 was the only MMM predicted to exceed the 2008 Robeson upper bound, but it does not exceed the 2019 upper bound established just one year after the study was published. The same researchers have used similar methods in more recent studies to theoretically screen many other MOF–polymer combinations for CO_2_ mixed gas separation performance. Highlights include the finding that MOFs with narrow pores sizes (3.75–5.12 Å), low BET surface areas (1000 m^2^·g^−1^), and moderate porosities (0.41–0.58) yield MMMs with high CO_2_ selectivity during CO_2_/N_2_ separation [175], and the finding that MOFs with narrow pores and low porosities generally lead to superior selective properties for MOF-based MMMs [176]. A more focused example is the study by Salahshoori et al. [177], where MD and MC are employed to simulate CO_2_ separation using an asymmetric MOF-based MMM composed of a polyethersulfone support layer and a ZIF-67/Pebax^®^ 1657 selective layer. Compared to pristine homopolymers, the hybrid membrane, and a symmetric ZIF-67/Pebax^®^ 1657 MMM, the asymmetric MOF-based MMM exhibited enhanced CO_2_ permeability and improved CO_2_ selectivity over both CH_4_ and N_2_. Notably, their simulated data were in good agreement with experimental results.

MD simulations have been employed to understand MOF–polymer compatibility in composite systems such as MMMs. For example, Semino et al. [195] used MD simulations and DFT calculations to develop a model that characterizes the microscopic interactions at a ZIF-8/PIM-1 interface. They found that the terminal amino groups of the ZIF-8 imidazole linkers and the cyano groups of PIM-1 had the most energetically favored interaction, with an average (NH)_ZIF-8_–(N)_PIM-1_ distance of 2.57 Å. Despite the proximity of these interactions, the interface was dominated by a region of high polymer rigidity and low polymer density, causing a ‘microvoid’ defect with an average length of 13 Å. Since models like the one developed by Semino et al. are transferrable to other MOF–polymer systems, they could be useful for screening MOF–polymer pairs with minimal formation of interfacial defects.

#### 5.3 Challenges and prospects regarding machine learning and computational studies

Although both ML and CM appear promising for advancing the future development of MOF-based MMMs for CO_2_ capture, some key shortcomings should be addressed. For ML approaches, the main challenge lies in the quality of the algorithmic fit to real data. Despite increasing complexity, algorithms often become limited in local extrema during learning and optimization or suffer from overfitting or underfitting data. This can be mitigated using alternative ML models or improving existing ones [190]. As ML rapidly evolves, novel methods are anticipated to become more effective in CO_2_ capture with MOF-based MMMs. In CM, a common issue is the assumption of perfect MOFs, polymers, and MMMs. Defects are often omitted, potentially leading to significant deviations from experimental data, especially with higher filler loadings that increase defect propensity, see section 3.5. Some MOFs exhibit structural flexibility that can impact gas transport, which is not always considered. Consequently, seemingly promising MOF-based MMMs identified via high-throughput simulations should undergo detailed experimental validation to address stability, compatibility, and model reliability concerns [114]. Another significant challenge in CM is the computational demand. The large number of MOFs screened makes CM inefficient and limits the ability to incorporate defects into existing models. With appropriate algorithms, ML can be much more efficient [196].

Notably, the application of ML in MMM research altogether is currently novel. A literature search via the Scopus search engine with ‘machine learning’ and ‘mixed matrix membrane’ in the article title, abstract, or keywords returns only 21 published studies, with just six related to mixed gas CO_2_ separation in MOF-based MMMs, all published between 2022 and 2024 (researched on July 24, 2024) [179,188,190,197–199]. This field of study remains nascent. With the rapid evolution of ML models, there appears to be a large untapped potential for ML in CO_2_ capture with MOF-based MMMs.

Finally, it is important to address the current discrepancies between ML and CM results. As exemplified, ML and CM may yield different optimal MOF and polymer conditions for mixed gas CO_2_ separation. This likely results from the current limitations of both methods; some ML models provide only a mediocre fit, while CM is constrained to ideal conditions. An extensive comparative study between ML and CM could elucidate their respective strengths, weaknesses, and complementary aspects to benefit the field of CO_2_ capture with MOF-based MMMs most efficiently in the future.

### 6 Considerations for large-scale integration

#### 6.1 Upscale production of MOF-based MMMs

Laboratory synthesis of MOF nano-fillers and their integration in MMMs can be relatively straightforward, fast, and efficient [200]. However, scaling up these processes presents a significant challenge for its use in practical applications [201]. Solvothermal MOF synthesis is expensive and highly energy-demanding, with many solvents being toxic or costly, making them less viable for commercialization. Additionally, conventional batch synthesis of MOFs suffers from low yield and purity at scale, and continuous production methods are still in their infancy, far from being commercially viable [202].

Many commonly reported MMM fabrication methods are also poorly scalable, including simple solution casting [94,203]. In contrast, HFMMMs offer greater potential for industrial commercialization because of their larger interfacial surface area and modular design. However, research on MOF-based HFMMMs remains relatively limited, comprising only a small fraction of the overall research efforts. Current research in MOF-based MMM development focuses predominantly on novel materials, as it has for more than a decade [94]. Since novel manufacturing methods explored at the laboratory scale are time-consuming, complex, reliant on specialized and expensive equipment, or involve solvents that may be governmentally regulated [204], greater emphasis should be placed on developing scalable fabrication approaches to realize the potential of MOF-based MMM for CO_2_ capture. As Dr. Richard W. Baker, founder of Membrane Technology and Research, stated [205]: “From the industrial perspective when you have an interesting new membrane material, you are at the beginning of the development process, not at the end.”

Recently, Chen et al. [206] demonstrated a hot-pressing method for fabricating MOF coatings. Unique to this method is that it is possible to create stable MOF coatings at a sub-meter scale by simply dispersing the metal precursor and the polymeric linker on top of a substrate sheet and hot-pressing the system to initiate integrated MOF formation. Even more recently, Kong et al. [207] used a similar method to produce graphene oxide (GO) membranes with incorporated covalent-organic frameworks (COFs) for dye filtration in aqueous environments. In their work, the hot-pressing step enhanced the interface between COF and GO for a preformed COF-based GO membrane on a nylon support. The preformed membrane was prepared on the nylon support through vacuum filtration of a COF–GO slurry. The concept of hot-pressing might be extended to MOF-based MMMs with further exploration of the methodology with complex precursor slurries to create stable, durable, and homogenous membranes at an industrially relevant scale.

#### 6.2 Extended performance evaluation

After overcoming the challenges of scaling up production, it is important to recognize that the conditions tested in the laboratory are often idealized compared to real-world CO_2_ capture applications. Despite the many MOF-based MMMs reported in the context of CO_2_ capture, most studies have relied exclusively on binary gas mixtures like CO_2_/N_2_ or CO_2_/CH_4_. Ideally, they should be tested under conditions that more closely resemble actual applications.

In literature, the gas mixtures most commonly referred to as promising candidates for large-scale CO_2_ capture include flue gas, natural gas, and biogas. Flue gases, the largest source of global CO_2_ emissions [208], contain not only CO_2_ and N_2_ but also co-existing H_2_O, O_2_, nitrogen oxides (NO*_x_*), and sulfur oxides (SO*_x_*) [209]. While raw natural gas and biogas mainly contain CO_2_ and CH_4_, they also encompass smaller fractions of N_2_, H_2_O, and trace contaminants such as H_2_S, SO_2_, NH_3_, and VOCs [6,210]. An increasing number of publications focus on MOF-based MMMs for H_2_S/CH_4_ and NH_3_/CH_4_ separation to mitigate pollutant emission control [211], but it is rarely reported from the perspective of CO_2_ capture. Currently, extended performance evaluations of MOF-based MMMs for CO_2_ capture, if any, mainly focus on reporting permeability and selectivity metrics across extended pressure or temperature ranges and testing the performance stability over extended periods of operation.

**6.2.1 Moisture.** The impact of moisture is commonly reported in MOF studies, and its effect on CO_2_ separation performance has been examined in numerous studies on polymer membranes [212–231]. Yet, the subject remains more rarely explored in MOF-based MMM research. For many MOF adsorbents, small amounts of water can impair CO_2_ adsorption properties by strongly interacting with adsorption sites, promoting competitive co-adsorption and impairing CO_2_ capacity. Extensive adsorption of water molecules can even disarrange the MOF framework by hydrolyzing weak coordination bonds, particularly for carboxylate linkers, which cause irreversible structure degradation and porosity loss [232–233]. Hence, an expensive drying step to remove most moisture can be necessary for the successful implementation of MOF-based MMMs in most industrial processes [234]. Thus, MOFs should be stable enough to withstand structure collapse under high humidity. This warrants an intensification of research on moisture-resistant MOF-based MMMs for CO_2_ capture.

MOF-based CO_2_ adsorption studies report various degrees of performance reduction in the presence of moisture. Water has a smaller kinetic diameter (2.65 Å), and a higher dipole moment (1.85 Debye) compared to CO_2_ (0 Debye), for which MOFs with OMSs preferably bind with water over CO_2_. As a result, many OMS-containing MOFs (e.g., the M-MOF-74 series [235–237] and Cu-TDPAT [238]) are unsuitable for applications in high-humidity environments. The high density of OMSs on the channel pore surfaces of M-MOF-74, which gives rise to their exceptional CO_2_ selectivity in CO_2_/N_2_ and CO_2_/CH_4_ separations, also leads to strong interactions with water. Computational simulations have suggested that the insertion of 2,4,6-tri(4-pyridyl)-1,3,5-triazine (tpt) ligands into the pores of M-MOF-74 (M = Mg or Zn) could be a strategy to re-engineer the pore space. According to ideal adsorbed solution theory (IAST) and GCMC simulation data, the smaller pores resulting from the occupation of tpt ligands are predicted to reduce water uptake from a 0.15 bar CO_2_/H_2_O mixture with H_2_O mole fractions varying from 0.001% to 10% (corresponding to 0.0048–48% RH) by preventing the formation of water clusters inside the pores [239]. However, this prediction remains to be experimentally verified.

In many cases where MOFs are prepared with OMSs, the adverse impact of moisture on CO_2_ adsorption is negligible (e.g., MIL-101(Cr) [218–219], ZIF-8 [220–221], SGU-29 [222], ZIF-Cl [221], SALEM-2 [221], and CALF-20 [223]). Moreover, examples exist of OMS-containing MOFs showing moisture-enhanced CO_2_ adsorption at certain conditions (e.g., MIL-100(Fe) [224], UiO-66 [225], MIL-96(Al) [226], IRMOF-74-III-(CH_2_NH_2_)_2_ [53], PCN-250(Fe_2_Co), and PCN-250(Fe_3_) [227]). An experimental study by Pokhrel et al. [220] found that the effect of pre-adsorbed water on a polyethyleneimine (PEI) functionalized ZIF-8 led to a 1–2% suppression of CO_2_ uptake compared to dry conditions. In another study by Yang et al. [228], the CO_2_ uptake of 30 wt % PEI-functionalized ZIF-8 was enhanced from 1.4 mmol·g^−1^ under dry conditions to 1.7 mmol·g^−1^ under 50% RH. The effect of moisture on CO_2_ adsorption by HKUST-1 has been studied by several groups [219,224,229–230]. Findings suggest that CO_2_ adsorption uptake is promoted at a certain relative humidity range, with CO_2_ uptake decreasing again at higher humidity because of competition with H_2_O for adsorption sites. Some have identified this optimum at 4 wt % water [229] and others around 10% RH [224]. At high humidity, CO_2_ uptake decreases and is accompanied by a loss of structural stability [224,231].

In the context of post-combustion carbon capture, Mason et al. [240] experimentally screened 15 different adsorbents, including nine MOFs, for CO_2_ adsorption in a ternary gas mixture of CO_2_, N_2,_ and H_2_O. They found that mmen-Mg-MOF-74 (mmen = *N*,*N*′-dimethylethylenediamine) had a CO_2_ capacity of 4.2 mmol·g^−1^ in humid multicomponent gas, compared to 3.7 mmol·g^−1^ in single-component CO_2_ tests. In contrast, Mg-MOF-74 exhibited a CO_2_ capacity of just 0.5 mmol·g^−1^ in humid conditions [240]. A recent computational study identified the most promising of 3703 MOFs from the CoRE-MOF-2019 database for wet flue gas (CO_2_, N_2_, and H_2_O at 80% RH) CO_2_ capture. Using a multiscale modeling approach that integrated ML, DFT calculations, force field optimization, and GCMC simulations, they identified the top six candidates from their data set, namely HIMSAY, LUFQUZ, RIPNUB, VEJZEQ, BUSQEM, and EREFEN01 [241]. The authors highlighted two key features that seem to drive CO_2_ selectivity over H_2_O; these are (1) complementary charged sites that align with the atomic charges in CO_2_, and (2) parallel aromatic linkers separated by ≈7 Å, which promote strong π–π interactions with CO_2_.

While the moisture effect on MOF-based MMMs is infrequently studied, numerous significant results have been reported. In some cases, moisture negatively impacts CO_2_ permeability in MOF-based MMMs, such as in ZIF-8/Pebax^®^ 2533 [242]. However, moisture can also enhance CO_2_ permeability. For example, defect-engineered Ni-MOF nanosheets (denoted Ni-NS-8-6) embedded in a Pebax^®^ 1657 matrix showed an increase in CO_2_ permeability from 170.3 Barrer in dry conditions to 436.1 Barrer at 100% RH, with no significant change in CO_2_/N_2_ selectivity [243]. A recent study has shown that 100% RH can improve both CO_2_ permeability and CO_2_/N_2_ selectivity in MMMs containing amine-functionalized ZIF-L nano-fillers (with PEI or PAMAM) in a Pebax^®^ 2533 matrix, with stable performance observed over 72 h of continuous operation under humid conditions [244].

Only one study has explored the effect of various humidity levels on the CO_2_ separation performance of MOF-based MMMs. Qin et al. [245] fabricated SUM-9/Pebax^®^ 2533 and SUM-1/Pebax^®^ 2533 MMMs, which displayed differing trends in CO_2_/N_2_ separation performance as RH varied between 0, 30, 60, and 100%. For SUM-9/Pebax^®^ 2533 with a theoretical MOF pore size of 6.06 Å, increasing humidity promoted CO_2_ transport, improving CO_2_ permeability and CO_2_/N_2_ selectivity. In contrast, SUM-1/Pebax^®^ 2533 with a theoretical MOF pore size of 9.9 Å exhibited a nonlinear response to increasing humidity, with CO_2_ permeability and CO_2_/N_2_ selectivity peaking at 30% RH. The authors attributed these contrasting behaviors to differences in SUM pore structure, which affect the competition between H_2_O and CO_2_ molecules during transport [245].

**Figure 12:**
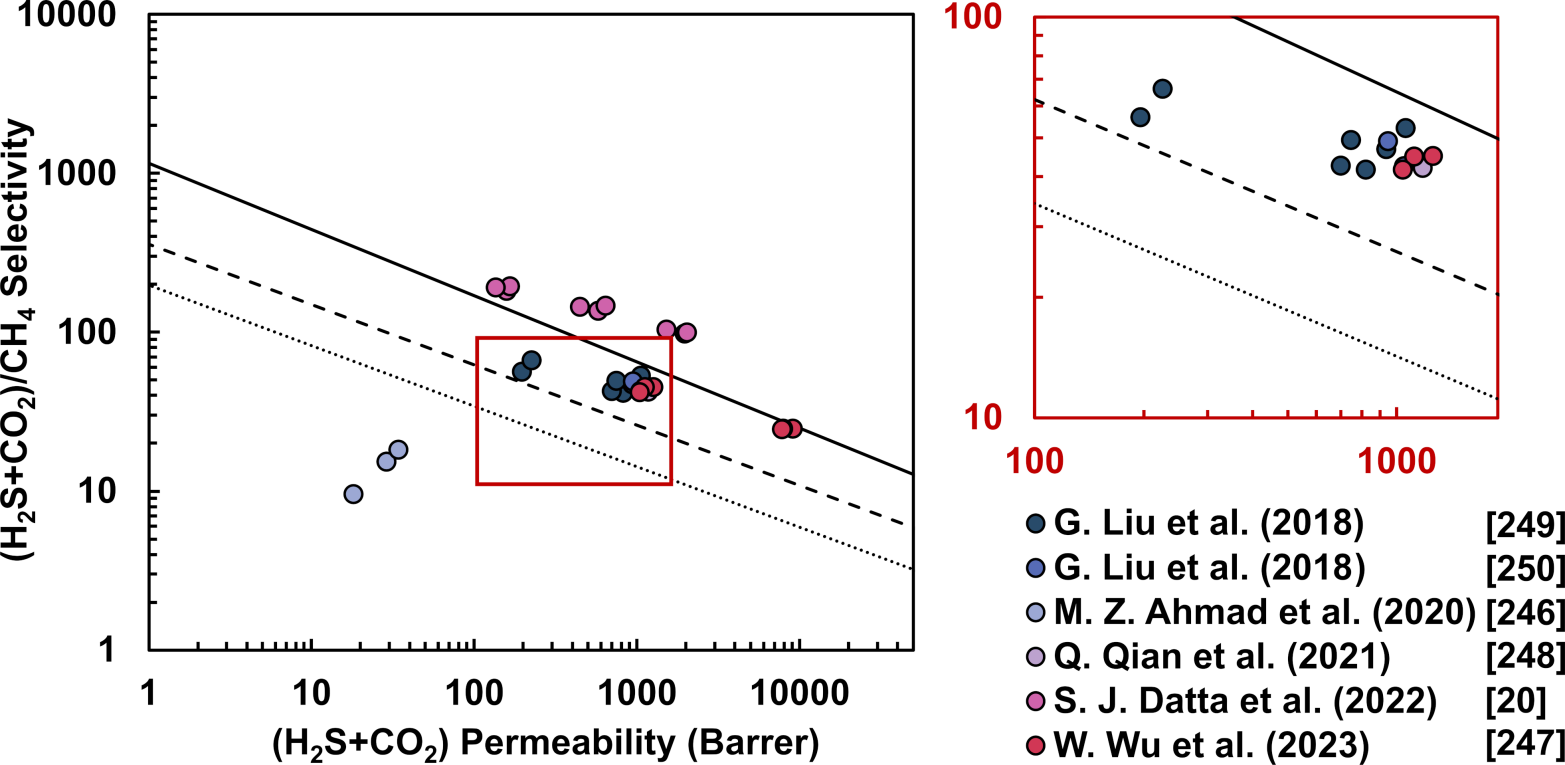
Selectivity/permeability characteristics of MOF-based MMMs for separation of ternary CO_2_/H_2_S/CH_4_ mixtures reported in the literature [20,246–250]. Lines indicate the 1991 (dotted) and 2008 (dashed) Robeson upper bound, as well as the 2019 upper bound proposed by Comesaña-Gándara and coworkers [13–14].

**6.2.2 Hydrogen sulfide.** For MOF-based MMMs to be viable for CO_2_ capture from natural gas or biogas streams, they should be tested under more complex conditions beyond binary CH_4_/CO_2_ separations. Natural gas and biogas contain H_2_S, which must be removed to meet pipeline specifications, so research on ternary H_2_S/CO_2_/CH_4_ separations is crucial. H_2_S has a kinetic diameter of 3.6 Å, between CO_2_ (3.3 Å) and CH_4_ (3.8 Å), and current studies are limited to molecular sieving mechanisms for separation. Reported feed gas compositions include various H_2_S/CO_2_/CH_4_ ratios (i.e., 1/9/90 [20], 2/18/80 [20], 5/5/90 [20], 5/30/65 [246], 13/27/60 [247], 27/13/60 [247], and 20/20/60 [247–250]), and all associated MOF-based MMM performance results reported to date are provided in Figure 12. The primary target in current studies is CH_4_ recovery, relying on co-removal of CO_2_ and H_2_S. A two-step separation system, that is, H_2_S/CO_2_/CH_4_ followed by H_2_S/CO_2_ separation, may be required for efficient CO_2_ capture.

#### 6.3 Handling large gas volumes

Industrial applicability requires the treatment of large volumes of bulk gas streams at low pressure. Nevertheless, gas separation is contingent on the partial pressure difference between the feed and permeate side of the membrane. Low-pressure gas streams necessitate the application of an external pressure difference across the MMM to ensure a reasonable CO_2_ permeability. This pressure difference can be obtained through compression of the feed gas stream, and likely requires a combination with a vacuum on the permeate side to enhance the pressure ratio. At large scale, however, this greatly limits the economic feasibility of MOF-based MMMs for CO_2_ separation from low-pressure streams [5]. At natural gas extraction facilities, including offshore applications, CO_2_ must be removed from high-pressure streams (40–70 bar) to on-site pipeline specification (from 10–30% to about 2–5% CO_2_) [5]. At such conditions, polymeric membranes and MOF-based MMMs hold the potential to compete with CO_2_ capture techniques such as amine scrubbing and conventional adsorbents [5,251–252]. Given the substantial volumes of natural gas extracted, achieving remarkably high permeability for CO_2_ is essential for implementing membranes to keep capital costs and spatial requirements low.

Recently, Jiang et al. [18] synthesized a MOF-based MMM for natural gas purification using NTU-88 nano-fillers in a 6DA-DAM matrix, featuring nanochannels with a pore aperture of 3.4 Å, which lies between the kinetic diameters of CO_2_ (3.3 Å) and CH_4_ (3.8 Å). They achieved a maximum CO_2_ permeability of 1140 Barrer with a CO_2_/CH_4_ selectivity of 34, positioning it near the 2019 upper bound. The MMM maintained stable separation performance over 200 h of operation, suggesting its potential for real-world application.

Based solely on reported CO_2_ permeability data, one of the MOF-based MMMs showing the greatest potential for large-scale CO_2_ capture from natural gas was developed by Tien-Binh and coworkers [253]. Similarly to the approach detailed in their later work [165] and described in section 4.2, they cross-linked PIM-1 polymer chain ends with –OH functional groups on the surface of Mg-MOF-74 fillers to obtain defect-free MMMs. At 20 wt % MOF loading, the MMM achieved a staggering CO_2_ permeability of 21269 Barrer and a CO_2_/CH_4_ selectivity of 19.1. The performance remained consistent across CO_2_ partial feed pressures (2–10 bar), suggesting its feasibility for a wide range of CO_2_/CH_4_ separation applications. Yin et al. [254] also prepared a PIM-1-based MMM, namely a MUF-15/PIM-1 flat sheet at 15 wt % MUF-15 loading achieving a CO_2_ permeability of 23400 Barrer. Several of the highest-performing MOF-based MMMs based on CO_2_ permeability are based on PIM-1 matrices [19,22,253–255]. To the best of our knowledge, the MOF-based MMM with the highest CO_2_ permeability reported to date was prepared by Khdhayyer and coworkers [19]. Solution casting of 47 vol % MIL-101 in a PIM-1 matrix followed by ethanol treatment yielded a MOF-based MMM with a CO_2_ permeability of an unprecedented 35600 Barrer with CO_2_/N_2_ selectivity of 15.3 and CO_2_/CH_4_ selectivity of 11.7. The as-cast membrane (not treated with ethanol) exhibited a CO_2_ permeability of 22000 Barrer and CO_2_/N_2_ and CO_2_/CH_4_ selectivities of 15.4 and 8.0, respectively. Mixed gas permeation experiments conducted after seven years of aging revealed that permeation performance decreased significantly (CO_2_ permeability 3500–3800 Barrer, CO_2_/N_2_ selectivity 25–27, CO_2_/CH_4_ selectivity 21–24), yet remained competitive to other reported MOF-based MMMs. None of these studies, however, addressed the effects of humidity or co-pollutants on separation performance.

**Figure 13:**
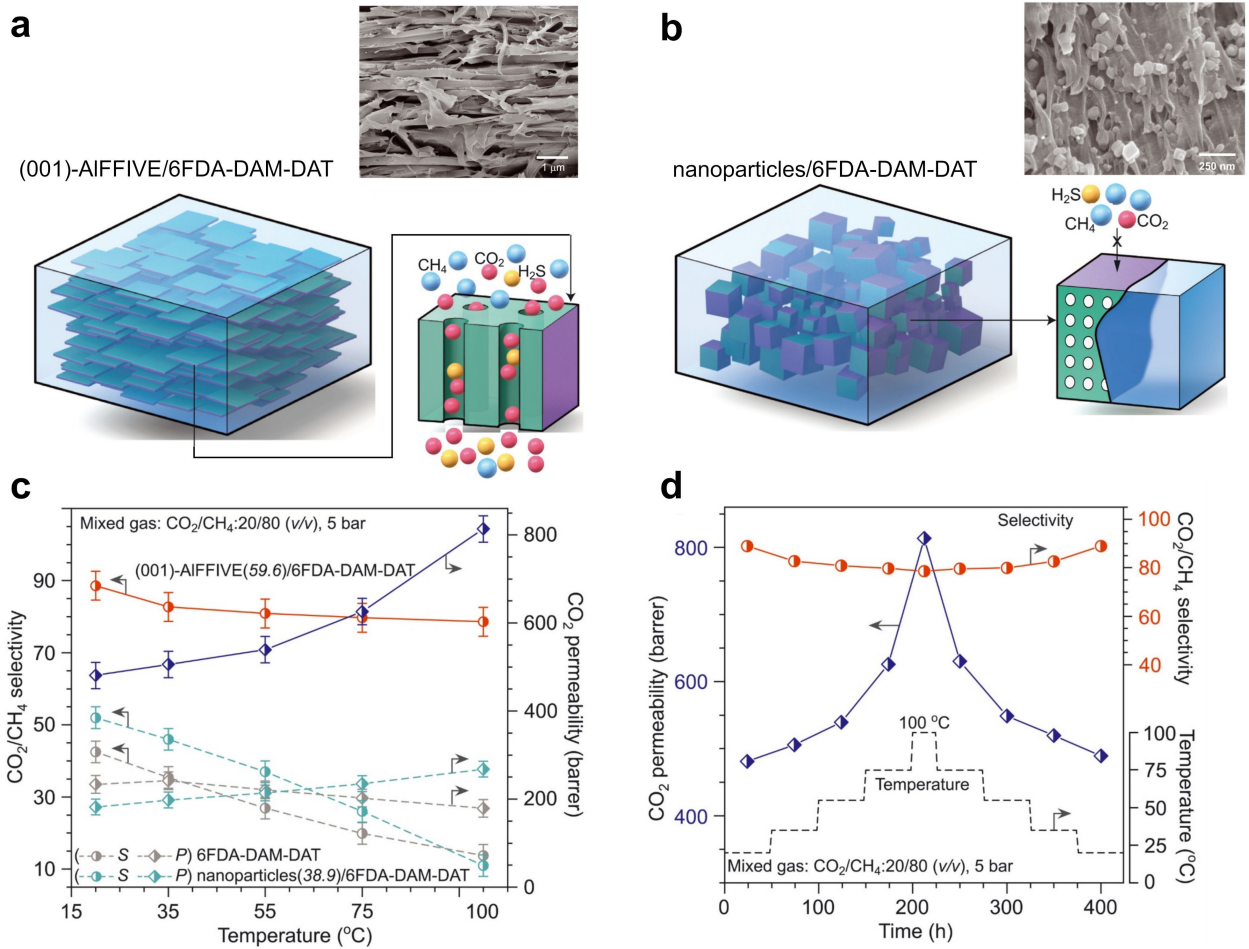
Schematic illustration and cross-section SEM images of (a) (001)-oriented membrane and (b) random fashion nanoparticle embedded in a polymer matrix. (c) Effects of temperature on CO_2_ permeability and CO_2_/CH_4_ selectivity. (d) Long-term stability and reversibility of CO_2_ permeability and CO_2_/CH_4_ selectivity under thermal stress in (001)-AlFFIVE(59.6)/6FDA-DAM-DAT membrane. From [20]. Reprinted with permission from AAAS. This content is not subject to CC BY 4.0.

Datta et al. [20] published a comprehensive paper demonstrating that tailoring MOF fillers into nanosheets can promote CO_2_ permeability and CO_2_/CH_4_ selectivity. Specifically, the authors prepared AlFFIVE-1-Ni nanosheets with a highly defined (001) crystallographic direction and used it in MMM fabrication with polyimides (6FDA-DAM, 6FDA-DAM-DAT, and 6FDA-DAT) matrices. At loadings of ≈60 wt % filler, performance metrics based on a 10/90 CO_2_/CH_4_ gas mixture aligned closely with the 2019 upper bound, namely a CO_2_ permeability of 435.9 Barrer and a CO_2_/CH_4_ selectivity of 84.7 for (001)-AlFFIVE-1-Ni/6FDA-DAM-DAT as well as a CO_2_ permeability of 129.8 Barrer and a CO_2_/CH_4_ selectivity of 141.8 for (001)-AlFFIVE-1-Ni/6FDA-DAT. The authors reported tests to showcase its feasibility in natural gas purification under practical working conditions, including tests of (1) varying binary feed compositions (CO_2_/CH_4_ = 10/90, 20/80, and 50/50), (2) co-presence of H_2_S (H_2_S/CO_2_/CH_4_ = 1/9/90, 2/18/80, and 20/20/60), (3) temperature dependence up to 100 °C, (4) pressure dependence up to 35 bar, (5) performance stability over 30 days of continuous operation, illustrated in Figure 13. The authors showed that the membrane could be prepared either through solution casting with slow solvent evaporation in the flat sheet configuration or through an ultrathin membrane coating by spin coating on top of a porous Al_2_O_3_ support.

In summary, future research should emphasize the practical challenges associated with real-world applications for a more comprehensive assessment of the feasibility of upscale production of MOF-based MMMs for industrial CO_2_ capture.

### 7 Outlook

Membrane technology has advanced significantly since Steinitzer [81] reported one of the first MMMs with inorganic filler for gas separation in 1912. In the past decade, MOF-based MMMs have emerged as a new generation of MMMs for CO_2_ capture. Although the research field is developing fast, several necessary leaps must be made if the technology is to reach a state of commercialization.

A great limitation that prevents MOF-based MMMs from reaching their full potential is defect formation at the MOF–polymer interface. As discussed throughout this review, there have been many different approaches to address this issue, each with varying success. In particular, deterministic defect engineering in the form of controlled MOF aggregation as discussed in section 3.5.4 through the study by Li et al. [123] appears promising. Unique to this method of preparing MOF-based MMMs is that the transport of the separated gas mixture is handled exclusively through MOFs in “gas transport highways”. In this way, the MMM exploits the full gas separation capabilities of the MOF, while leveraging the mechanical properties of the polymer membrane. Future research may benefit from utilizing similar approaches and creating advanced MOF networks within the MMM for optimal CO_2_ capture performance. In addition, this circumvents the issue of homogenous MOF distribution within the MMM, which remains a challenge for upscaling MOF-based MMM production.

The application of ML in CO_2_ capture using MOF-based MMMs is still in its infancy but holds tremendous potential for MOF-based MMM design. As Yao et al. [190] exemplified through their GA-optimized backpropagation network, ML already shows great promise in optimizing lab-scale MOF-based MMMs for mixed gas CO_2_ separation. Future efforts should focus on expanding ML applications to more complex CO_2_ capture scenarios, employing larger and more sophisticated data sets. In turn, this will improve the predictive capabilities of ML models for a broad set of CO_2_ capture processes in various contexts.

Similarly, CM with MD and MC simulations has shown potential in CO_2_ capture research. With a rapidly increasing computational power, such simulations will likely become increasingly advanced and useful. Thus, while future research in this field should focus on screening potential materials for advanced MOF-based MMMs, incorporating environmental intricacies and imperfections in the MOF–polymer system is of great relevance.

Furthermore, with more advanced ML models, it may become possible to incorporate effects from MOF–polymer interface enhancements, such as through MOF or polymer functionalization, or intermediate binding agents such as ionic liquids (section 3.5.6). Once such models have been established, large leaps within the fields are likely.

Although rarely discussed in the literature, the environmental humidity and other gas impurities at the application site for MOF-based MMMs have a major impact on CO_2_ capture efficiency as illustrated by Qin and coworkers [245]. As these effects are not an issue for laboratory studies, they are often omitted. However, these effects are crucial for large-scale application of MOF-based MMMs for CO_2_ capture. It is pivotal that such effects are studied in-depth for future large-scale applications of these systems.

Overall, it would be useful to test and report the mixed gas CO_2_ separation performance of MOF-based MMMs under industrially relevant conditions. This would also provide more relevant data for CM and ML, which may further accelerate advancements in the field.

## Conclusion

In the past decade, MMMs with MOF fillers have gained increasing attention for CO_2_ capture applications due to the combined advantages of polymeric membranes with the tunable and exceptional CO_2_ separation characteristics of MOFs from mixed gas. Through selected examples, this review identifies challenges and opportunities in MOF-based MMM design, development, and CO_2_ separation performance. A broad overview of relevant aspects in the field of CO_2_ capture by MOF-based MMMs is provided, and current trends as well as further areas of investigation are highlighted.

Current reports reveal that integrating compatible MOF fillers in MMMs can overcome the inherent permeability–selectivity trade-off for polymeric membranes for CO_2_ separation from CH_4_ and N_2_. Recent advancements in the field include computational and experimental approaches to understand and enhance MOF–polymer compatibility and interfacial adhesion during the design and development stages. Despite recent scientific progress, MOF-based MMMs with ideal properties and economic feasibility for commercialization have yet to be demonstrated. With the recent progress in CM and ML, these methods may prove to be invaluable tools for identifying industrially relevant MOF-based MMMs for CO_2_ capture. Finally, it is stressed that it will be highly relevant to test and report CO_2_ capture performance under industrially relevant conditions to prepare and advance the field for industrial applications.

To advance technological readiness, interdisciplinary research collaborations including computational scientists, experimental scientists, and process engineers are crucial to rapidly advance the field and promote the industrial implementation of MOF-based MMMs for CO_2_ capture in targeted applications.

## Supporting Information

File 1 Literature and data used for Figure 10.

## Data Availability

Data sharing is not applicable as no new data was generated or analyzed in this study.

## References

[1] Yoro K O, Daramola M O, Rahimpour M R, Farsi M, Makarem M A (2020). Chapter 1 - CO2 Emission Sources, Greenhouse Gases, and the Global Warming Effect. Advances in Carbon Capture.

[2] Fernández J R, Garcia S, Sanz-Pérez E S (2020). Ind Eng Chem Res.

[3] Mikkelsen M, Jørgensen M, Krebs F C (2010). Energy Environ Sci.

[4] Kanehashi S, Scholes C A (2020). Front Chem Sci Eng.

[5] Muthukumaraswamy Rangaraj V, Wahab M A, Reddy K S K, Kakosimos G, Abdalla O, Favvas E P, Reinalda D, Geuzebroek F, Abdala A, Karanikolos G N (2020). Front Chem (Lausanne, Switz).

[6] Aghel B, Behaein S, Alobaid F (2022). Fuel.

[7] Liu R-S, Shi X-D, Wang C-T, Gao Y-Z, Xu S, Hao G-P, Chen S, Lu A-H (2021). ChemSusChem.

[8] Surmi A (2019). Process Integration and Optimization of CO2 Removal from Natural Gas Using Cryogenic Distillation System.

[9] Chuah C Y, Kim K, Lee J, Koh D-Y, Bae T-H (2020). Ind Eng Chem Res.

[10] Ji Y, Zhang M, Guan K, Zhao J, Liu G, Jin W (2019). Adv Funct Mater.

[11] Fujikawa S, Selyanchyn R, Kunitake T (2021). Polym J.

[12] Zhu B, Jiang X, He S, Yang X, Long J, Zhang Y, Shao L (2020). J Mater Chem A.

[13] Robeson L M (2008). J Membr Sci.

[14] Comesaña-Gándara B, Chen J, Bezzu C G, Carta M, Rose I, Ferrari M-C, Esposito E, Fuoco A, Jansen J C, McKeown N B (2019). Energy Environ Sci.

[15] Katare A, Kumar S, Kundu S, Sharma S, Kundu L M, Mandal B (2023). ACS Omega.

[16] Galizia M, Chi W S, Smith Z P, Merkel T C, Baker R W, Freeman B D (2017). Macromolecules.

[17] He Z, Kumar Reddy K S, Karanikolos G, Wang K, Basile A, Favvas E P (2018). Chapter 5 - CO2/CH4 Separation (Natural Gas Purification) by Using Mixed Matrix Membranes. Current Trends and Future Developments on (Bio-) Membranes.

[18] Jiang F, Zhao J, Wan J, Zheng B, Chang I-Y, Duan J, Jin W (2024). J Membr Sci.

[19] Khdhayyer M, Bushell A F, Budd P M, Attfield M P, Jiang D, Burrows A D, Esposito E, Bernardo P, Monteleone M, Fuoco A (2019). Sep Purif Technol.

[20] Datta S J, Mayoral A, Murthy Srivatsa Bettahalli N, Bhatt P M, Karunakaran M, Carja I D, Fan D, Mileo P G M, Semino R, Maurin G (2022). Science.

[21] Xin Q, Cao X, Huang D, Li S, Zhang X, Xuan G, Wei M, Zhang L, Ding X, Zhang Y (2022). Green Chem Eng.

[22] Sun Y, Geng C, Zhang Z, Qiao Z, Zhong C (2022). J Membr Sci.

[23] Demir H, Aksu G O, Gulbalkan H C, Keskin S (2022). Carbon Capture Sci Technol.

[24] Seoane B, Coronas J, Gascon I, Benavides M E, Karvan O, Caro J, Kapteijn F, Gascon J (2015). Chem Soc Rev.

[25] Jusoh N, Yeong Y F, Chew T L, Lau K K, Shariff A M (2016). Sep Purif Rev.

[26] Sumida K, Rogow D L, Mason J A, McDonald T M, Bloch E D, Herm Z R, Bae T-H, Long J R (2012). Chem Rev.

[27] Lin R, Villacorta Hernandez B, Ge L, Zhu Z (2018). J Mater Chem A.

[28] Kitao T, Zhang Y, Kitagawa S, Wang B, Uemura T (2017). Chem Soc Rev.

[29] Daglar H, Erucar I, Keskin S (2021). Mater Adv.

[30] Furukawa H, Cordova K E, O’Keeffe M, Yaghi O M (2013). Science.

[31] Kinoshita Y, Matsubara I, Higuchi T, Saito Y (1959). Bull Chem Soc Jpn.

[32] Cheong V F, Moh P Y (2018). Mater Sci Technol.

[33] Khosravi T, Omidkhah M, Kaliaguine S, Rodrigue D (2017). Can J Chem Eng.

[34] Pu Y, Yang Z, Wee V, Wu Z, Jiang Z, Zhao D (2022). J Membr Sci.

[35] Wang H, Ding Y, Ning M, Yu M, Zheng W, Ruan X, Xi Y, Dai Y, Liu H, He G (2023). Sep Purif Technol.

[36] Farrusseng D, Aguado S, Pinel C (2009). Angew Chem, Int Ed.

[37] Simon-Yarza T, Mielcarek A, Couvreur P, Serre C (2018). Adv Mater (Weinheim, Ger).

[38] Wang H, Lustig W P, Li J (2018). Chem Soc Rev.

[39] Kumar P, Deep A, Kim K-H (2015). TrAC, Trends Anal Chem.

[40] Farajzadeh R, Glasbergen G, Karpan V, Mjeni R, Boersma D M, Eftekhari A A, Casquera Garcia A, Bruining J (2022). J Cleaner Prod.

[41] Wang S, Tountas A A, Pan W, Zhao J, He L, Sun W, Yang D, Ozin G A (2021). Small.

[42] Wajid U, Cappiello C, Plebani P, Pernici B, Mehandjiev N, Vitali M, Gienger M, Kavoussanakis K, Margery D, Perez D G (2016). IEEE Trans Cloud Comput.

[43] Du R, Li C, Liu Q, Fan J, Peng Y (2022). Bioresour Technol.

[44] Ding M, Flaig R W, Jiang H-L, Yaghi O M (2019). Chem Soc Rev.

[45] Piscopo C G, Loebbecke S (2020). ChemPlusChem.

[46] Younas M, Rezakazemi M, Daud M, Wazir M B, Ahmad S, Ullah N, Inamuddin, Ramakrishna S (2020). Prog Energy Combust Sci.

[47] Li H, Wang K, Sun Y, Lollar C T, Li J, Zhou H-C (2018). Mater Today.

[48] Petit C (2018). Curr Opin Chem Eng.

[49] Yu J, Xie L-H, Li J-R, Ma Y, Seminario J M, Balbuena P B (2017). Chem Rev.

[50] Zhuang W, Yuan D, Liu D, Zhong C, Li J-R, Zhou H-C (2012). Chem Mater.

[51] Taylor M K, Runčevski T, Oktawiec J, Bachman J E, Siegelman R L, Jiang H, Mason J A, Tarver J D, Long J R (2018). J Am Chem Soc.

[52] Kong X, Scott E, Ding W, Mason J A, Long J R, Reimer J A (2012). J Am Chem Soc.

[53] Flaig R W, Osborn Popp T M, Fracaroli A M, Kapustin E A, Kalmutzki M J, Altamimi R M, Fathieh F, Reimer J A, Yaghi O M (2017). J Am Chem Soc.

[54] Shi Y, Liang B, Lin R-B, Zhang C, Chen B (2020). Trends Chem.

[55] Ghanbari T, Abnisa F, Wan Daud W M A (2020). Sci Total Environ.

[56] Mehio N, Dai S, Jiang D-e (2014). J Phys Chem A.

[57] Breck D W (1974). Zeolite Molecular Sieves.

[58] Berdichevsky E K, Downing V A, Hooper R W, Butt N W, McGrath D T, Donnelly L J, Michaelis V K, Katz M J (2022). Inorg Chem.

[59] Yao X, Cordova K E, Zhang Y-B (2022). Small Struct.

[60] Li G, Kujawski W, Válek R, Koter S (2021). Int J Greenhouse Gas Control.

[61] Luo F, Yan C, Dang L, Krishna R, Zhou W, Wu H, Dong X, Han Y, Hu T-L, O’Keeffe M (2016). J Am Chem Soc.

[62] Gao J, Qian X, Lin R-B, Krishna R, Wu H, Zhou W, Chen B (2020). Angew Chem, Int Ed.

[63] Wu H, Simmons J M, Srinivas G, Zhou W, Yildirim T (2010). J Phys Chem Lett.

[64] Valenzano L, Civalleri B, Chavan S, Palomino G T, Areán C O, Bordiga S (2010). J Phys Chem C.

[65] Queen W L, Hudson M R, Bloch E D, Mason J A, Gonzalez M I, Lee J S, Gygi D, Howe J D, Lee K, Darwish T A (2014). Chem Sci.

[66] Queen W L, Brown C M, Britt D K, Zajdel P, Hudson M R, Yaghi O M (2011). J Phys Chem C.

[67] Wan Y, Miao Y, Zhong R, Zou R (2022). Nanomaterials.

[68] Fracaroli A M, Furukawa H, Suzuki M, Dodd M, Okajima S, Gándara F, Reimer J A, Yaghi O M (2014). J Am Chem Soc.

[69] Graham T (1866). Philos Trans R Soc London.

[70] Zhao S, Zhang G, Shen W, Wang X, Liu F (2020). J Appl Phys.

[71] Burns R L, Koros W J (2003). Macromolecules.

[72] Kayvani Fard A, McKay G, Buekenhoudt A, Al Sulaiti H, Motmans F, Khraisheh M, Atieh M (2018). Materials.

[73] Daramola M O, Aransiola E F, Ojumu T V (2012). Materials.

[74] Rezakazemi M, Sadrzadeh M, Matsuura T (2018). Prog Energy Combust Sci.

[75] Trickett C A, Helal A, Al-Maythalony B A, Yamani Z H, Cordova K E, Yaghi O M (2017). Nat Rev Mater.

[76] Cheng Y, Ying Y, Japip S, Jiang S-D, Chung T-S, Zhang S, Zhao D (2018). Adv Mater (Weinheim, Ger).

[77] Carreon M A, Li S, Falconer J L, Noble R D (2008). J Am Chem Soc.

[78] Moore T T, Koros W J (2005). J Mol Struct.

[79] Wang M, Wang Z, Zhao S, Wang J, Wang S (2017). Chin J Chem Eng.

[80] Aroon M A, Ismail A F, Matsuura T, Montazer-Rahmati M M (2010). Sep Purif Technol.

[81] Steinitzer F (1912). Gummi-Ztg.

[82] Kulprathipanja S (2003). Ann N Y Acad Sci.

[83] Kulprathipanja S, Neuzil R W, Li N N (1992). Separation of Gases by Means of Mixed Matrix Membranes. US patent.

[84] Mahajan R, Koros W J (2000). Ind Eng Chem Res.

[85] Yehia H A T E M, Pisklak T J, Ferraris J P, Balkus K J, Musselman I H (2004). Methane Facilitated Transport Using Copper (II) Biphenyl Dicarboxylate-Triethylenediamine/Poly(3-Acetoxyethylthiophene) Mixed Matrix Membranes. Abstracts of Papers of the American Chemical Society;.

[86] Chen Z, Zhang Y, Song Q, Ma L, Lv Y (2022). STAR Protoc.

[87] Carreon M, Dahe G, Feng J, Venna S R, Carreon M A (2016). Mixed Matrix Membranes for Gas Separation Applications. Membrane Science and Technology.

[88] Wyart Y, Tamime R, Siozade L, Baudin I, Glucina K, Deumié C, Moulin P (2014). J Membr Sci.

[89] Yu S, Li C, Zhao S, Chai M, Hou J, Lin R (2024). Nanoscale.

[90] Tanvidkar P, Appari S, Kuncharam B V R (2022). Rev Environ Sci Bio/Technol.

[91] Maleh M S, Raisi A (2023). Colloids Surf, A.

[92] Basu S, Cano-Odena A, Vankelecom I F J (2010). J Membr Sci.

[93] Sunder N, Fong Y Y, Bustam M A, Suhaimi N H (2022). Polymers (Basel, Switz).

[94] Nasir R, Mukhtar H, Man Z, Mohshim D F (2013). Chem Eng Technol.

[95] Burmann P, Zornoza B, Téllez C, Coronas J (2014). Chem Eng Sci.

[96] Kulak H, Thür R, Vankelecom I F J (2022). Membranes.

[97] Sasikumar B, Bisht S, Arthanareeswaran G, Ismail A F, Othman M H D (2021). Sep Purif Technol.

[98] Sutrisna P D, Hou J, Zulkifli M Y, Li H, Zhang Y, Liang W, D'Alessandro D M, Chen V (2018). J Mater Chem A.

[99] Zhu H, Jie X, Wang L, Kang G, Liu D, Cao Y (2018). J Energy Chem.

[100] Mubashir M, Yeong Y F, Chew T L, Lau K K (2019). Sep Purif Technol.

[101] Hou J, Sutrisna P D, Zhang Y, Chen V (2016). Angew Chem, Int Ed.

[102] Zhu H, Wang L, Jie X, Liu D, Cao Y (2016). ACS Appl Mater Interfaces.

[103] Chen H Z, Thong Z, Li P, Chung T-S (2014). Int J Hydrogen Energy.

[104] Mai Z, Liu D (2019). Cryst Growth Des.

[105] Lin R-B, Xiang S, Zhou W, Chen B (2020). Chem.

[106] Gopalsamy K, Fan D, Naskar S, Magnin Y, Maurin G (2024). ACS Appl Eng Mater.

[107] Yuan S, Zou L, Qin J-S, Li J, Huang L, Feng L, Wang X, Bosch M, Alsalme A, Cagin T (2017). Nat Commun.

[108] Yang Y, Fernández-Seriñán P, Imaz I, Gándara F, Handke M, Ortín-Rubio B, Juanhuix J, Maspoch D (2023). J Am Chem Soc.

[109] Rose M, Weber D, Lotsch B V, Kremer R K, Goddard R, Palkovits R (2013). Microporous Mesoporous Mater.

[110] Shekhah O, Belmabkhout Y, Chen Z, Guillerm V, Cairns A, Adil K, Eddaoudi M (2014). Nat Commun.

[111] Stock N, Biswas S (2012). Chem Rev.

[112] Sabetghadam A, Liu X, Benzaqui M, Gkaniatsou E, Orsi A, Lozinska M M, Sicard C, Johnson T, Steunou N, Wright P A (2018). Chem – Eur J.

[113] Katayama Y, Bentz K C, Cohen S M (2019). ACS Appl Mater Interfaces.

[114] Keskin S, Alsoy Altinkaya S (2019). Computation.

[115] Friebe S, Diestel L, Knebel A, Wollbrink A, Caro J (2016). Chem Ing Tech.

[116] Winarta J, Meshram A, Zhu F, Li R, Jafar H, Parmar K, Liu J, Mu B (2020). J Polym Sci (Hoboken, NJ, U S).

[117] Moore T T, Mahajan R, Vu D Q, Koros W J (2004). AIChE J.

[118] Ding X, Li X, Zhao H, Wang R, Zhao R, Li H, Zhang Y (2018). Chin J Chem Eng.

[119] Goh S H, Lau H S, Yong W F (2022). Small.

[120] Han Y, Ho W S W (2018). Chin J Chem Eng.

[121] Ban Y, Li Z, Li Y, Peng Y, Jin H, Jiao W, Guo A, Wang P, Yang Q, Zhong C (2015). Angew Chem, Int Ed.

[122] Yang T, Xiao Y, Chung T-S (2011). Energy Environ Sci.

[123] Li C, Qi A, Ling Y, Tao Y, Zhang Y-B, Li T (2023). Sci Adv.

[124] Song S, Zhao M, Guo Z, Ren Y, Wang J, Liang X, Pu Y, Wang S, Ma H, Wang X (2023). J Membr Sci.

[125] Chi W S, Sundell B J, Zhang K, Harrigan D J, Hayden S C, Smith Z P (2019). ChemSusChem.

[126] Vanherck K, Koeckelberghs G, Vankelecom I F J (2013). Prog Polym Sci.

[127] Lee H, Chi W S, Lee M J, Zhang K, Edhaim F, Mizrahi Rodriguez K, DeWitt S J A, Smith Z P (2022). Adv Funct Mater.

[128] Carja I-D, Tavares S R, Shekhah O, Ozcan A, Semino R, Kale V S, Eddaoudi M, Maurin G (2021). ACS Appl Mater Interfaces.

[129] Gadipelli S, Guo Z (2014). Chem Mater.

[130] Lai W-H, Zhuang G-L, Tseng H-H, Wey M-Y (2019). J Membr Sci.

[131] Song Q, Nataraj S K, Roussenova M V, Tan J C, Hughes D J, Li W, Bourgoin P, Alam M A, Cheetham A K, Al-Muhtaseb S A (2012). Energy Environ Sci.

[132] Lin R, Ge L, Diao H, Rudolph V, Zhu Z (2016). ACS Appl Mater Interfaces.

[133] Li G, Kujawski W, Tonkonogovas A, Knozowska K, Kujawa J, Olewnik-Kruszkowska E, Pedišius N, Stankevičius A (2022). Chem Eng Res Des.

[134] Liu B, Li D, Yao J, Sun H (2020). Sep Purif Technol.

[135] Li M, Zhang X, Zeng S, bai L, Gao H, Deng J, Yang Q, Zhang S (2017). RSC Adv.

[136] Li H, Tuo L, Yang K, Jeong H-K, Dai Y, He G, Zhao W (2016). J Membr Sci.

[137] Lu J, Zhang X, Xu L, Zhang G, Zheng J, Tong Z, Shen C, Meng Q (2021). Membranes.

[138] Casado-Coterillo C, Fernández-Barquín A, Zornoza B, Téllez C, Coronas J, Irabien Á (2015). RSC Adv.

[139] Penczek P A, Jensen G J (2010). Resolution Measures in Molecular Electron Microscopy. Methods in Enzymology.

[140] Mohamed M A, Jaafar J, Ismail A F, Othman M H D, Rahman M A, Hilal N, Ismail A F, Matsuura T (2017). Fourier Transform Infrared (FTIR) Spectroscopy. Membrane Characterization.

[141] Kwan A H, Mobli M, Gooley P R, King G F, Mackay J P (2011). FEBS J.

[142] Bunaciu A A, Udriştioiu E G, Aboul-Enein H Y (2015). Crit Rev Anal Chem.

[143] Wu C, Guo H, Liu X, Zhang B (2022). Sep Purif Technol.

[144] Hodoroaba V-D, Hodoroaba V-D, Unger W E S, Shard A G (2020). Energy-Dispersive X-Ray Spectroscopy (EDS). Characterization of Nanoparticles.

[145] Vernon-Parry K D (2000). III-Vs Review.

[146] Nuhnen A, Klopotowski M, Tanh Jeazet H B, Sorribas S, Zornoza B, Téllez C, Coronas J, Janiak C (2020). Dalton Trans.

[147] Sasikumar B, Arthanareeswaran G (2022). Sep Purif Technol.

[148] Stevie F A, Donley C L (2020). J Vac Sci Technol, A.

[149] Sinha P, Datar A, Jeong C, Deng X, Chung Y G, Lin L-C (2019). J Phys Chem C.

[150] Jean Y C, Van Horn J D, Hung W-S, Lee K-R (2013). Macromolecules.

[151] Franken L E, Grünewald K, Boekema E J, Stuart M C A (2020). Small.

[152] Maia R A, Louis B, Gao W, Wang Q (2021). React Chem Eng.

[153] Car A, Stropnik C, Peinemann K-V (2006). Desalination.

[154] Nik O G, Chen X Y, Kaliaguine S (2012). J Membr Sci.

[155] Feijani E A, Mahdavi H, Tavassoli A (2018). New J Chem.

[156] Basu S, Cano-Odena A, Vankelecom I F J (2011). Sep Purif Technol.

[157] Ge B, Xu Y, Zhao H, Sun H, Guo Y, Wang W (2018). Materials.

[158] Zornoza B, Seoane B, Zamaro J M, Téllez C, Coronas J (2011). ChemPhysChem.

[159] O’Neill L D, Zhang H, Bradshaw D (2010). J Mater Chem.

[160] Chui S S-Y, Lo S M-F, Charmant J P H, Orpen A G, Williams I D (1999). Science.

[161] Du M, Li L, Li M, Si R (2016). RSC Adv.

[162] Wong-Ng W, Levin I, Kaduk J A, Espinal L, Wu H (2016). J Alloys Compd.

[163] He Y, Zhou W, Qian G, Chen B (2014). Chem Soc Rev.

[164] Habib N, Durak O, Zeeshan M, Uzun A, Keskin S (2022). J Membr Sci.

[165] Tien-Binh N, Rodrigue D, Kaliaguine S (2018). J Membr Sci.

[166] He S, Zhu B, Jiang X, Han G, Li S, Lau C H, Wu Y, Zhang Y, Shao L (2022). Proc Natl Acad Sci U S A.

[167] Lin R, Ge L, Liu S, Rudolph V, Zhu Z (2015). ACS Appl Mater Interfaces.

[168] Wei J, Chu X, Sun X-Y, Xu K, Deng H-X, Chen J, Wei Z, Lei M (2019). InfoMat.

[169] Carleo G, Cirac I, Cranmer K, Daudet L, Schuld M, Tishby N, Vogt-Maranto L, Zdeborová L (2019). Rev Mod Phys.

[170] Roscher R, Bohn B, Duarte M F, Garcke J (2020). IEEE Access.

[171] Tamai Y, Tanaka H, Nakanishi K (1996). Mol Simul.

[172] Bai G, Pan Y, Zhang Y, Li Y, Wang J, Wang Y, Teng W, Jin G, Geng F, Cao J (2023). Food Chem.

[173] Zhou S, Shi J, Liu S, Li G, Pei F, Chen Y, Deng J, Zheng Q, Li J, Zhao C (2023). Nature.

[174] Altintas C, Keskin S (2019). ACS Sustainable Chem Eng.

[175] Daglar H, Keskin S (2019). Adv Theory Simul.

[176] Daglar H, Aydin S, Keskin S (2022). Sep Purif Technol.

[177] Salahshoori I, Babapoor A, Seyfaee A (2022). Polym Bull.

[178] Choi R Y, Coyner A S, Kalpathy-Cramer J, Chiang M F, Campbell J P (2020). Transl Vis Sci Technol.

[179] Guan J, Huang T, Liu W, Feng F, Japip S, Li J, Wu J, Wang X, Zhang S (2022). Cell Rep Phys Sci.

[180] Adir O, Poley M, Chen G, Froim S, Krinsky N, Shklover J, Shainsky-Roitman J, Lammers T, Schroeder A (2020). Adv Mater (Weinheim, Ger).

[181] Taber C S, Timpone R J (1996). Computational Modeling; Quantitative Applications in the Social Sciences.

[182] Frenkel D, Smit B, Frenkel D, Smit B (2023). Molecular Dynamics Simulations. Understanding Molecular Simulation.

[183] Maurin G, Rouquerol F, Rouquerol J, Sing K S W (2014). Modelling of Physisorption in Porous Solids. Adsorption by Powders and Porous Solids.

[184] Moghadam P Z, Li A, Wiggin S B, Tao A, Maloney A G P, Wood P A, Ward S C, Fairen-Jimenez D (2017). Chem Mater.

[185] Burner J, Luo J, White A, Mirmiran A, Kwon O, Boyd P G, Maley S, Gibaldi M, Simrod S, Ogden V (2023). Chem Mater.

[186] Chung Y G, Haldoupis E, Bucior B J, Haranczyk M, Lee S, Zhang H, Vogiatzis K D, Milisavljevic M, Ling S, Camp J S (2019). J Chem Eng Data.

[187] Thornton A W, Freeman B D, Robeson L M (2012). Polymer Gas Separation Membrane Database.

[188] Wang J, Tian K, Li D, Chen M, Feng X, Zhang Y, Wang Y, Van der Bruggen B (2023). Sep Purif Technol.

[189] Schonlau M, Zou R Y (2020). The Stata Journal.

[190] Yao L, Zhang Z, Li Y, Zhuo J, Chen Z, Lin Z, Liu H, Yao Z (2024). Sep Purif Technol.

[191] Zou J, Han Y, So S-S, Livingstone D J (2009). Overview of Artificial Neural Networks. Artificial Neural Networks: Methods and Applications.

[192] Shapiro J, Paliouras G, Karkaletsis V, Spyropoulos C D (2001). Genetic Algorithms in Machine Learning. Machine Learning and Its Applications: Advanced Lectures.

[193] Lundberg S M, Lee S-I, Guyon I, Luxburg U V, Bengio S (2017). A Unified Approach to Interpreting Model Predictions. Advances in Neural Information Processing Systems.

[194] Maxwell J (1954). A Treatise on Electricity and Magnetism.

[195] Semino R, Ramsahye N A, Ghoufi A, Maurin G (2016). ACS Appl Mater Interfaces.

[196] Demir H, Daglar H, Gulbalkan H C, Aksu G O, Keskin S (2023). Coord Chem Rev.

[197] Demir H, Keskin S (2024). Macromol Mater Eng.

[198] Alizamir M, Keshavarz A, Abdollahi F, Khosravi A, Karagöz S (2023). Sep Purif Technol.

[199] Zhang Z, Cao X, Geng C, Sun Y, He Y, Qiao Z, Zhong C (2022). J Membr Sci.

[200] Zhuang J-L, Ceglarek D, Pethuraj S, Terfort A (2011). Adv Funct Mater.

[201] He Q, Zhan F, Wang H, Xu W, Wang H, Chen L (2022). Mater Today Sustainability.

[202] Paul T, Juma A, Alqerem R, Karanikolos G, Arafat H A, Dumée L F (2023). J Environ Chem Eng.

[203] Liu S, Liu G, Chen G, Liu G, Jin W (2023). Curr Opin Chem Eng.

[204] Sheng M, Dong S, Qiao Z, Li Q, Yuan Y, Xing G, Zhao S, Wang J, Wang Z (2021). J Membr Sci.

[205] Hennessy J (2017). Nat Mater.

[206] Chen Y, Li S, Pei X, Zhou J, Feng X, Zhang S, Cheng Y, Li H, Han R, Wang B (2016). Angew Chem, Int Ed.

[207] Kong G, Pang J, Tang Y, Fan L, Sun H, Wang R, Feng S, Feng Y, Fan W, Kang W (2019). J Mater Chem A.

[208] Usubharatana P, McMartin D, Veawab A, Tontiwachwuthikul P (2006). Ind Eng Chem Res.

[209] Van Den Hende S, Vervaeren H, Boon N (2012). Biotechnol Adv.

[210] Muñoz R, Meier L, Diaz I, Jeison D (2015). Rev Environ Sci Bio/Technol.

[211] Duma Z, Makgwane P R, Masukume M, Swartbooi A, Rambau K, Mehlo T, Mavhungu T (2024). Mater Today Sustainability.

[212] Liu Z, Du Z, Zou W, Mi J, Li H, Wang Y, Zhang C (2013). RSC Adv.

[213] Lasseuguette E, Carta M, Brandani S, Ferrari M-C (2016). Int J Greenhouse Gas Control.

[214] Chand S, Pal A, Das M C (2018). Chem – Eur J.

[215] Li Y-Z, Wang G-D, Lu S, Xu F, Zhang H, Sui Y, Hou L (2024). Chem Eng J.

[216] Chen C, Jiang Q, Xu H, Lin Z (2019). Ind Eng Chem Res.

[217] Kim S, Lee Y M (2013). Curr Opin Chem Eng.

[218] Liu Q, Ning L, Zheng S, Tao M, Shi Y, He Y (2013). Sci Rep.

[219] Pirngruber G D, Hamon L, Bourrelly S, Llewellyn P L, Lenoir E, Guillerm V, Serre C, Devic T (2012). ChemSusChem.

[220] Pokhrel J, Bhoria N, Anastasiou S, Tsoufis T, Gournis D, Romanos G, Karanikolos G N (2018). Microporous Mesoporous Mater.

[221] Hu J, Liu Y, Liu J, Gu C (2017). Fuel.

[222] Datta S J, Khumnoon C, Lee Z H, Moon W K, Docao S, Nguyen T H, Hwang I C, Moon D, Oleynikov P, Terasaki O (2015). Science.

[223] Nguyen T T T, Balasubramaniam B M, Fylstra N, Huynh R P S, Shimizu G K H, Rajendran A (2024). Ind Eng Chem Res.

[224] Soubeyrand-Lenoir E, Vagner C, Yoon J W, Bazin P, Ragon F, Hwang Y K, Serre C, Chang J-S, Llewellyn P L (2012). J Am Chem Soc.

[225] Jajko G, Kozyra P, Gutiérrez-Sevillano J J, Makowski W, Calero S (2021). Chem – Eur J.

[226] Benoit V, Chanut N, Pillai R S, Benzaqui M, Beurroies I, Devautour-Vinot S, Serre C, Steunou N, Maurin G, Llewellyn P L (2018). J Mater Chem A.

[227] Chen Y, Qiao Z, Huang J, Wu H, Xiao J, Xia Q, Xi H, Hu J, Zhou J, Li Z (2018). ACS Appl Mater Interfaces.

[228] Yang F, Ge T, Zhu X, Wu J, Wang R (2022). Sep Purif Technol.

[229] Yazaydın A Ö, Benin A I, Faheem S A, Jakubczak P, Low J J, Willis R R, Snurr R Q (2009). Chem Mater.

[230] Liu J, Wang Y, Benin A I, Jakubczak P, Willis R R, LeVan M D (2010). Langmuir.

[231] Gul-E-Noor F, Jee B, Pöppl A, Hartmann M, Himsl D, Bertmer M (2011). Phys Chem Chem Phys.

[232] Jasuja H, Huang Y-g, Walton K S (2012). Langmuir.

[233] Low J J, Benin A I, Jakubczak P, Abrahamian J F, Faheem S A, Willis R R (2009). J Am Chem Soc.

[234] Chidambaram A, Le D H, Navarro J A R, Stylianou K C (2021). Appl Mater Today.

[235] Kizzie A C, Wong-Foy A G, Matzger A J (2011). Langmuir.

[236] Yu J, Balbuena P B (2013). J Phys Chem C.

[237] Liu J, Tian J, Thallapally P K, McGrail B P (2012). J Phys Chem C.

[238] Zhang Z, Li Z, Li J (2012). Langmuir.

[239] Suh B L, Lee S, Kim J (2017). J Phys Chem C.

[240] Mason J A, McDonald T M, Bae T-H, Bachman J E, Sumida K, Dutton J J, Kaye S S, Long J R (2015). J Am Chem Soc.

[241] Kancharlapalli S, Snurr R Q (2023). ACS Appl Mater Interfaces.

[242] Nafisi V, Hägg M-B (2014). J Membr Sci.

[243] Zhu W, Wang L, Cao H, Guo R, Wang C (2023). J Membr Sci.

[244] Qin Z, Ma Y, Du W, Wei J, Song J, Fan X, Yao L, Yang L, Zhuang Y, Jiang W (2024). Sep Purif Technol.

[245] Qin Z, Feng X, Yin D, Xin B, Jin Z, Deng Y, Yang L, Yao L, Jiang W, Liu C (2023). Ind Eng Chem Res.

[246] Ahmad M Z, Peters T A, Konnertz N M, Visser T, Téllez C, Coronas J, Fila V, de Vos W M, Benes N E (2020). Sep Purif Technol.

[247] Wu W-N, Mizrahi Rodriguez K, Roy N, Teesdale J J, Han G, Liu A, Smith Z P (2023). ACS Appl Mater Interfaces.

[248] Qian Q, Wright A M, Lee H, Dincă M, Smith Z P (2021). Chem Mater.

[249] Liu G, Chernikova V, Liu Y, Zhang K, Belmabkhout Y, Shekhah O, Zhang C, Yi S, Eddaoudi M, Koros W J (2018). Nat Mater.

[250] Liu G, Cadiau A, Liu Y, Adil K, Chernikova V, Carja I-D, Belmabkhout Y, Karunakaran M, Shekhah O, Zhang C (2018). Angew Chem, Int Ed.

[251] Sandru M, Sandru E M, Ingram W F, Deng J, Stenstad P M, Deng L, Spontak R J (2022). Science.

[252] Veziri C, Labropoulos A, Karanikolos G N, Kanellopoulos N K (2015). Recent Developments in Membrane Technologies for CO2 Separation. Small-Scale Gas to Liquid Fuel Synthesis.

[253] Tien-Binh N, Vinh-Thang H, Chen X Y, Rodrigue D, Kaliaguine S (2016). J Membr Sci.

[254] Yin H, Alkaş A, Zhang Y, Zhang Y, Telfer S G (2020). J Membr Sci.

[255] Ozcan A, Fan D, Datta S J, Diaz-Marquez A, Semino R, Cheng Y, Joarder B, Eddaoudi M, Maurin G (2024). Sci Adv.

